# A novel tetratricopeptide repeat protein, WHITE TO GREEN1, is required for early chloroplast development and affects RNA editing in chloroplasts

**DOI:** 10.1093/jxb/erx383

**Published:** 2017-11-11

**Authors:** Fei Ma, Yingchun Hu, Yan Ju, Qianru Jiang, Zhijun Cheng, Quan Zhang, Sodmergen

**Affiliations:** 1 Key Laboratory of Ministry of Education for Cell Proliferation and Differentiation, College of Life Sciences, Peking University, China; 2 National Key Facility for Crop Gene Resources and Genetic Improvement, Institute of Crop Science, Chinese Academy of Agricultural Sciences, China

**Keywords:** *Arabidopsis thaliana*, chloroplast, early development, MORFs, plastid gene, PPR, RNA editing, TPR, virescent

## Abstract

The chloroplast is essential for plant photosynthesis and production, but the regulatory mechanism of chloroplast development is still elusive. Here, a novel gene, *WHITE TO GREEN1* (*WTG1*), was identified to have a function in chloroplast development and plastid gene expression by screening Arabidopsis leaf coloration mutants. *WTG1* encodes a chloroplast-localized tetratricopeptide repeat protein that is expressed widely in Arabidopsis cells. Disruption of *WTG1* suppresses plant growth, retards leaf greening and chloroplast development, and represses photosynthetic gene expression, but complemented expression of *WTG1* restored a normal phenotype. Moreover, WTG1 protein is associated with the organelle RNA editing factors MORF8 and MORF9, and RNA editing of the plastid *petL-5* and *ndhG-50* transcripts was affected in *wtg1* mutants. These results indicate that WTG1 affects both transcriptional and posttranscriptional regulation of plastid gene expression, and provide evidence for the involvement of a tetratricopeptide repeat protein in chloroplast RNA editing in Arabidopsis.

## Teaser

WTG1 is involved in early chloroplast development and may function in RNA editing by enhancing the function of RNA editosomes in Arabidopsis

## Introduction

Chloroplast biogenesis is crucial for higher plant growth and development, on which all life ultimately depends (Waters and Langdale, 2009). The process by which the proplastids develop into functional chloroplasts is rather complex, and numerous proteins have been reported to be involved. These proteins are either nuclear-encoded or plastid-encoded, and their correct assembly and proper function require coordination of the two organelles at the transcriptional, RNA processing, translational, and post-translational levels (Pogson and Albrecht, 2011). When this coordination is undermined, the plant may exhibit severely affected phenotypes. In Arabidopsis, the nuclear-encoded sigma (*sig*) factors mediate plastid gene transcription directly, and chloroplast biogenesis is significantly delayed in *sig2* or *sig6* null mutants (Ishizaki *et al.*, 2005, Chi *et al.*, 2010). The plastid transcriptionally active chromosome (pTAC) 3/10/12 genes, also encoded by nuclear genes, are required for plastid transcription, and loss of any of them results in lethality without exogenous carbon sources (Pfalz *et al.*, 2006, Yagi *et al.*, 2012, Pfalz and Pfannschmidt, 2013). The rice nuclear gene *VIRESCENT2* encodes a guanylate kinase, and the *Osvir2* mutation disrupts the chloroplast translation machinery, causing a chlorotic phenotype (Iba *et al.*, 1991, Sugimoto *et al.*, 2004, Sugimoto *et al.*, 2007). In maize (*Zea mays*), the DNA- and RNA-binding protein ZmWHY2, which is encoded by a nuclear gene, is essential for chloroplast development, and the mutant allele causes a severe phenotype of albino seedlings lacking plastid ribosomes (Prikryl *et al.*, 2008).

Among the factors present in the chloroplast that are encoded by nuclear genes, the most notable ones are the helical repeat proteins: the pentatricopeptide repeat (PPR) and tetratricopeptide repeat (TPR) proteins (Schmitz-Linneweber and Small, 2008, Stern *et al.*, 2010, Shikanai and Fujii, 2013). The function of PPRs has been well characterized. Chloroplast-localized PPR proteins have been demonstrated to be involved in regulating plastid RNA editing (Lurin *et al.*, 2004, Kotera *et al.*, 2005, Tillich *et al.*, 2005, Okuda *et al.*, 2006, 2007, 2009, 2010, Chateigner-Boutin *et al.*, 2008, Hammani *et al.*, 2009, Yu *et al.*, 2009, Bentolila *et al.*, 2012, Hayes *et al.*, 2013, Yagi *et al.*, 2013, Kindgren *et al.*, 2015, Wagoner *et al.*, 2015, Yap *et al.*, 2015), RNA splicing (Schmitz-Linneweber *et al.*, 2006, Ichinose *et al.*, 2012), RNA processing (Fisk *et al.*, 1999, Meierhoff *et al.*, 2003, Hattori *et al.*, 2007), RNA stability (Yamazaki *et al.*, 2004, Beick *et al.*, 2008), and translation (Williams and Barkan, 2003, Tavares-Carreón *et al.*, 2008).

RNA editing is a posttranscriptional process that converts specific cytidines (C) to uridines (U) in mitochondria and plastids (Covello and Gray, 1989, Hiesel *et al.*, 1989, Hoch *et al.*, 1991). Considerable evidence has shown that PPR proteins play crucial roles in RNA editing as the sequence-specific *trans*-factors that recognize editing sites (Okuda *et al.*, 2006, Okuda and Shikanai, 2012). In addition, members of the RIP (RNA-editing factor interacting protein)/MORF (multiple organellar RNA editing factor) family, the ORRM (organelle RNA recognition motif-containing) family, and the RanBP2-type zinc finger protein family, as well as one tetrapyrrole biosynthetic enzyme, have also been identified as factors involved in editing Arabidopsis plastid RNA transcripts (Takenaka *et al.*, 2012, Sun *et al.*, 2013, 2015, Zhang *et al.*, 2014, Shi *et al.*, 2015, 2016*a*, 2016*b*). In Arabidopsis, the RIP/MORF family contains 10 members, including RIP1/MORF8, which targets both plastids and mitochondria, and RIP2/MORF2 and RIP9/MORF9, which target plastids (Takenaka *et al.*, 2012). Defects in any of these factors affect the majority of RNA editing sites in plastids (Bentolila *et al.*, 2012; Takenaka *et al.*, 2012).

The TPR proteins, which are similar to PPR proteins, contain tandem repeats of 34 amino acids (one amino acid fewer than the number in a PPR motif) (Hirano *et al.*, 1990, Sikorski *et al.*, 1990). Repeated PPR and TPR motifs form a super-helix with a central groove that bonds a target molecule (Das *et al.*, 1998, Delannoy *et al.*, 2007); it has been suggested that PPR domains may bond preferably to RNAs (Delannoy *et al.*, 2007), while TPR domains may bond preferably to host proteins (Das *et al.*, 1998, D’Andrea and Regan, 2003). TPR proteins in the plastid always play an important role in chloroplast gene expression (Hu *et al.*, 2014), protein turnover (Park *et al.*, 2007), photosystem assembly and repair (Park *et al.*, 2007, Heinnickel *et al.*, 2016), chlorophyll biosynthesis, and thylakoid membrane biogenesis (Schottkowski *et al.*, 2009). For example, the *Chlamydomonas reinhardtii* CGL71 is a TPR protein integral to chloroplast thylakoid membranes, and the *cgl71* mutant cannot perform normal photosynthesis (Heinnickel *et al.*, 2016). The orthologous protein of CGL71 in Arabidopsis, Pale Yellow Green7 (PYG7), is required for photosystem I accumulation, and deletion of *Pyg7* results in alterations in leaf coloration and severely reduced growth rates (Stöckel *et al.*, 2006). SLOW-GREENING1 (SG1), a chloroplast-localized TPR protein in Arabidopsis, has been demonstrated to be involved in regulating the expression of genes associated with photosynthesis, chlorophyll biosynthesis, and chloroplast development, while its mutant displays a slow-greening phenotype (Hu *et al.*, 2014).

Here, a novel TPR protein was identified to affect chloroplast development in Arabidopsis and designated WHITE TO GREEN1 (WTG1). T-DNA insertion mutant plants (*wtg1*) have an albinic and dwarf phenotype, retarded chloroplast development, disturbed expression of chloroplast-related genes, and significantly lowered editing rates at two (*petL-5* and *ndhG-50*) of 43 plastid RNA editing sites. WTG1 did not bind the *cis*-elements of *petL-5* and *ndhG-50* sites; instead, WTG1 interacted with plastid-localized MORFs. These findings suggest that WTG1 is required for chloroplast development and may function in RNA editing in Arabidopsis by enhancing the function of RNA editosomes.

## Materials and methods

All primers are listed in Supplementary Table S1 at *JXB* online.

### Plant material and culture

*Arabidopsis thaliana* ecotype Columbia (Col-0) was used as the wild type in this work. The T-DNA insertion alleles in *WTG1* (*wtg1-1*, *wtg1-2*, and *wtg1-3*) were obtained from the Arabidopsis Biological Resource Center (ABRC) stock center (SALK_071495, SALK_006120, and SALK_015164, respectively). All seeds were cultivated on agar plates containing 50% Murashige and Skoog medium (Murashige, 1962) and transplanted to soil in a greenhouse at 22 °C with a 16 h/8 h light (100 μmol m^−2^ s^−1^)/dark cycle.

### Genetic analysis

Total genomic DNA was isolated as described previously (Edwards *et al.*, 1991). Gene-speciﬁc primers, together with the Lba1 primer, were used to test the T-DNA insertion lines. Homozygous plants were identiﬁed and used for the following phenotypic analyses.

### RNA extraction and RT-PCR

Total RNA was extracted from tissue samples using Trizol reagent (Invitrogen) according to the manufacturer’s instructions. To synthesize cDNA, total RNA was reverse-transcribed using the primeScript 1st Strand cDNA Synthesis Kit (TaKaRa). Primer sets L2/R2 and L2/LBa1 were used to determine the abundance of the *WTG1* mRNA transcript in the wild-type, wild-type/*wtg1* heterozygote, and *wtg1* homozygote plants.

To investigate the expression pattern of *WTG1*, total RNA isolated from roots, stems, leaves, inflorescences, and siliques was subjected to RT-PCR using the L1/R1 primers. *UBQ5* was amplified as a control using the UBQ5F/UBQ5R primers.

To detect the expression levels of chloroplast-related genes, quantitative real-time PCR (qRT-PCR) analysis was performed. qRT-PCR amplification was carried out in a LightCycler® 480 Real-Time PCR System. Primer sets are listed in Supplementary Table S1. Relative quantification of gene expression data was performed as described by Livak and Schmittgen (2001).

### RNA-seq analysis

Total RNA was isolated from leaves of wild-type, *wtg1*, and complemented lines at the 18- and 50-day-old stages. mRNA enriched from total RNA was fragmented and reverse-transcribed using random hexamer primers. The library was then constructed and sequenced using an Illumina HiSeq 4000 (BIOPIC-Beijing). Clean reads were aligned to the *A. thaliana* genome (TAIR 10.0). Levels of gene expression were calculated using the RPKM (reads per kilobase transcript per million reads) method.

The significance of differentially expressed genes (DEGs) was determined through iSeq (http://iseq.cbi.pku.edu.cn) by fold change less than 0.33 or greater than 3. Gene ontology analysis was performed by DAVID 6.7 (Huang *et al.*, 2009).

### Plasmid construction

#### For genetic complementation and subcellular localization

The *WTG1* coding sequence (CDS) without the stop codon was PCR-amplified from wild-type cDNA with 53080cds_L1/53080link_R1 primers as a WTG1-link fragment. *green fluorescent protein* (*GFP*) was amplified from the pGreen0029-DUO1-DIPS-GFP-NOS plasmid with linkGFP_L1/linkGFP_R1 primers to yield a link-GFP fragment. Using the WTG1-link and link-GFP fragments as a template, 53080cds_L1/linkGFP_R1 primers were used to generate a WTG1-GFP fragment, which was digested with *Bam*HI and *Sal*I and ligated into the *Bam*HI*-Sal*I site of pWM101 to yield a pWM101-35S-WTG1-GFP vector. A *35S*-*GFP* construct was used as a negative control.

#### For protein expression

The full-length *WTG1* coding region was amplified from genomic DNA (ecotype Col-0) using *Bam*HI-53080-F/53080-*Eco*RI-R primers, after which the product was cut by *Bam*HI and *Eco*RI and cloned into the pGEX-4T-1 vector (GE Healthcare) within the *Bam*HI and *Eco*RI sites to yield a pGEX-WTG1 vector.

#### For yeast two-hybrid assay

For the yeast two-hybrid assay, the CDSs of WTG1, OTP82, MORF2, MORF8, MORF9, ORRM1, OZ1, and PPO1 were ampliﬁed from wild-type cDNA using gene-speciﬁc primer sets. Full-length WTG1 was ligated into pGBKT7 (BD) to generate a pBD-WTG1 plasmid, while full-length OTP82, MORF2, MORF8, MORF9, ORRM1, OZ1, and PPO1 were cloned into pGADT7 (AD) to yield pAD-OTP82, pAD-ORF2, pAD-MORF8, pAD-MORF9, pAD-ORRM1, pAD-OZ1, and pAD-PPO1 plasmids, respectively (see Supplementary Table S1 for primer sequences and cloning sites).

#### For bimolecular fluorescence complementation assay

For the bimolecular fluorescence complementation (BiFC) assay, MORF8, MORF9, and WTG1 were cloned into binary BiFC vectors pSPYNE173 and pSPYCE (M) to produce MORF8/9-eYNE and WTG1-eYCE plasmids (Waadt and Kudla, 2008), respectively (see Supplementary Table S1 for primer sequences and cloning sites).

### Genetic complementation

The constructed pWM101-35S-WTG1-GFP vector was introduced into *Agrobacterium tumefaciens* strain GV3101 by electroporation and transformed into homozygous mutants (wtg*1-1/wtg1-1*) by ﬂoral dipping (Clough and Bent, 1998). The homozygous *wtg1-1* mutants rescued by *35S-WTG1-GFP* transgenic plants were conﬁrmed by hygromycin selection and genotyping.

### Detection of chlorophyll

Total chlorophyll was determined according to the method described by Lichtenthaler and Wellburn (1983). Extracts were obtained from 50 mg of fresh tissue from 18-, 35- and 50-day-old plants and homogenized in 100 ml of 80% acetone. Spectrophotometric quantiﬁcation was carried out in a U-1800 spectrophotometer (Hitachi).

### Transmission electron microscopy

We harvested 50-day-old leaves from wild-type plants, *wtg1* homozygotes, and complemented lines (all three lines), as well as 18-day-old albino leaves and 35-day-old pale green leaves from *wtg1* mutants. Samples (approximately 1 mm^2^) were cut with a new blade, fixed with 4% glutaraldehyde for 4 h at room temperature, and post-fixed in 2% osmium tetroxide for 1 h at room temperature. The samples were rinsed twice in 20 mM phosphate buffer (pH 7.0) and dehydrated in 30 min steps in a graded series of ethanol concentrations (10%, 30%, 50%, 70%, 85%, 95%, and two changes of 100%). We transferred the dehydrated samples to a gradient mixture of Spurr’s embedding medium and 100% ethanol (1:3, 1:1, and 3:1 respectively, v/v; 4 h in each concentration) at room temperature. Next, the samples were transferred into pure Spurr’s medium and incubated in a mixer for 24 h at room temperature. Finally, the samples were allowed to sit at 65 °C for 20 h to complete the embedding. Ultrathin sections of the samples were cut with a diamond knife on an ultramicrotome (Leica UC7) and collected on single-mesh copper grids. The sections were stained with 2% aqueous uranyl acetate and lead citrate before being viewed using an electron microscope (FEI Tecnai G^2^ 20).

### Subcellular localization

The constructed pWM101-35S-WTG1-GFP and pWM101-35S-GFP vectors were introduced separately into *A. tumefaciens* strain GV3101. Wild-type plants were stably transformed with the pWM101-35S-WTG1-GFP and pWM101-35S-GFP transformants via the floral dipping method (Clough and Bent, 1998). Transgenic plants were conﬁrmed by hygromycin selection and genotyping, after which mesophyll cells were obtained. The GFP signal was observed using a DMI 6000 B microscope (Leica).

### RNA editing analysis

Total RNA was extracted from plants at specific stages and reverse-transcribed as templates.

#### Bulk sequencing

Thirty-four distinct RNA editing sites were ampliﬁed and sequenced by speciﬁc primers (Cai *et al.*, 2009). RNA editing rates were estimated by the relative heights of nucleotide peaks in the analyzed sequence.

#### Pyrosequencing

To verify the C (unedited) to U (edited) ratios at the *petL-5* and *ndhG-50* sites, we performed PCR with petLpyro-F/petLpyro-R (biotinylated) and ndhGLpyro-F/ndhGpyro-R (biotinylated) primers, respectively. PCR products were converted into single-stranded DNA templates using a PyroMark Q24 Vacuum Workstation (Qiagen) according to the manufacturer’s instructions. Pyrosequencing reactions were performed in a PyroMark Q24 Advanced System (Qiagen). C:U ratios were analyzed using PyroMark Q24 Advanced Software (Qiagen).

### RNA electrophoretic mobility-shift assay

RNA electrophoretic mobility-shift assay (REMSA) was performed as described previously (Lin and Xu, 2012). Briefly, to prepare the template, equimolar oligonucleotides were mixed, heat-denatured, and annealed in Taq DNA polymerase buffer (NEB). Next, the template (0.5 μM final concentration) was used for *in vitro* RNA transcription by T7 RNA polymerase (NEB) following the manufacturer’s instructions. The templates were digested with RNase-free DNase I (TaKaRa), after which RNA probes were purified with the RNeasy Mini Kit (Qiagen) and then incubated with WTG1 protein at 30 °C for 30 min in a 20 μl system containing 20 mM Tris-acetate (pH 7.9), 50 mM potassium acetate, 10 mM magnesium, 2.5 mM dithiothreitol, 500 μg/ml BSA, and 40 units of RNase inhibitor. To prevent non-specific binding, 500 μg/ml BSA was added to the reaction system. RNA–protein complexes were resolved on a 1.5% agarose gel and detected by GelRed staining using the methods described by Lin and Xu (2012).

### RNA immunoprecipitation

*35S-WTG1-GFP* transgenic seedlings (18 days old) were fixed with 1% formaldehyde. Chloroplast isolation and subsequent immunoprecipitation of specific RNA–protein complexes were performed as described previously (Ketcham *et al.*, 1984, Terzi and Simpson, 2009). Anti-GFP (AbCam) and Dynabeads® ProteinG (Thermo Fisher) beads were used for immunoprecipitation. Next, RNA was isolated and reverse-transcribed. For real-time PCR, 1 μl of cDNA was loaded as the template. The negative control consisted of the sample without an antibody.

### Yeast two-hybrid assay

The pGBKT7-WTG1 plasmid was cotransformed into yeast strain AH109 with pGADT7-OTP82, pGADT7-MORF2, pGADT7-MORF8, pGADT7-MORF9, pGADT7-ORRM1, pGADT7-OZ1, or pGADT7-PPO1. Yeast transformation, screening for positive clones, and subsequent assays were performed according to the manufacturer’s instructions (Clontech). Plasmids without *WTG1* or other editing factors were used as negative controls.

### Bimolecular fluorescence complementation assay

*Agrobacterium tumefaciens* strain GV3103 was co-transformed with MORF8-eYNE and WTG1-eYCE, or MORF9-eYNE and WTG1-eYCE. The MORF2-eYNE and WTG1-eYCE combination was used as the negative control, whereas the MORF2-eYNE and MORF9-eYCE combination was used as the positive control. Each combination was introduced into *A. tumefaciens* strain GV3103 by electroporation. The transformants were then used to inﬁltrate *Nicotiana benthamiana* leaves as described previously (Waadt and Kudla, 2008). After 48 h, the infiltrated leaves were subjected to confocal imaging analysis using an LSM 710 NLO laser scanning confocal microscope (Zeiss).

### Accession numbers

Sequence data from this report can be found in the Arabidopsis Genome Initiative or GenBank/EMBL databases under accession numbers At5g53080 (WTG1) and AP000423 (Arabidopsis plastid genome).

## Results

### *WTG1* is a nuclear single-copy gene required for normal seedling growth and pigmentation

**Fig. 1.**
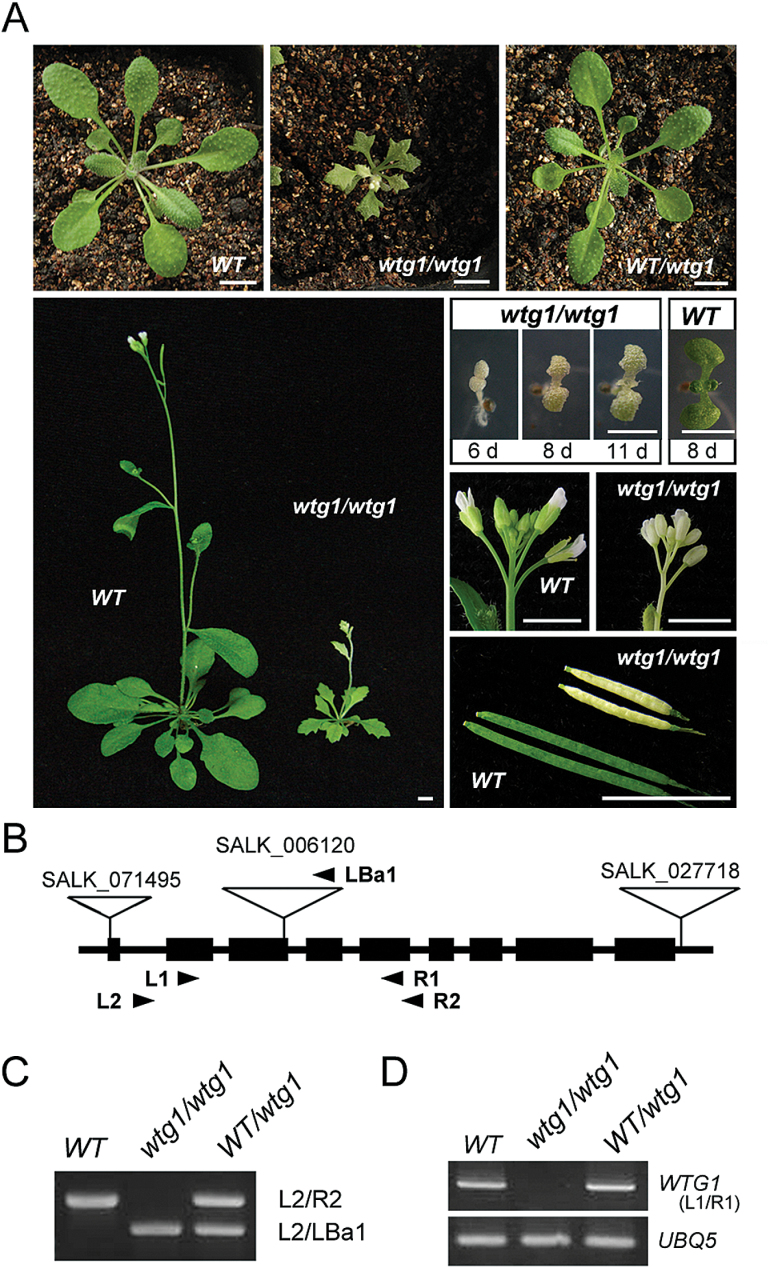
A *WTG1* T-DNA insertional mutant exhibits a phenotype of dwarfism and albinism. (A) Phenotypes of wild-type (WT), *wtg1* homozygotes, and heterozygotes. (B) Schematic representation of the *WTG1* gene with exons shown as black rectangles and T-DNA insertions shown as triangles. The primers used for RT-PCR are indicated by arrowheads. (C) Identiﬁcation of WT, *wtg1* homozygotes, and *wtg1/+* heterozygotes using insertion site analysis. (D) RT-PCR confirmation of *wtg1* mutants. *UBQ5* served as the loading control.

To study the mechanism of chloroplast development, we screened abnormal leaf coloration mutants from T-DNA insertion Arabidopsis lines (Alonso and Stepanova, 2003) and identified a mutant (SALK_006120) termed *White-To-Green1-1* (*wtg1-1*). The homozygous mutants (*wtg1-1/wtg1-1*) germinated as dwarf and albino seedlings with serrated leaves, which, interestingly, turned green as they matured; these plants were fertile and had short siliques with a reduced seed set (Fig. 1A; Supplementary Fig. S1). Self-pollinated heterozygotes produced 607 offspring plants, among which the ratio of albino plants to green plants was 157:450 [χ^2^ (3:1)=0.241<χ^2^_0.85_]. These results indicate that the defective leaf coloration phenotype of *wtg1-1* mutants was regulated by a recessive allele and inherited by Mendelian genetic principles.

**Fig. 2.**
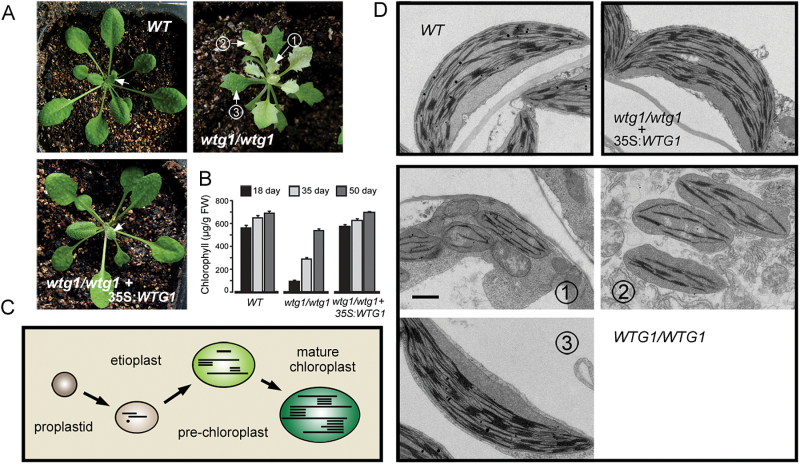
Loss of *WTG1* postpones leaf greening and delays chloroplast development. (A) The *wtg1* homozygotes show a delayed greening phenotype, and *Pro35S:WTG1* restored the wild-type (WT) appearance. Arrows indicate the sites sampled for transmission electron microscopy. (B) Chlorophyll content in WT, *wtg1*, and complemented lines at different growth stages. Data are presented as mean±SD of triplicates. (C) The mode pattern of chloroplast biogenesis. (D) Transmission electron micrographs of chloroplasts from WT, *wtg1*, and complemented plants. Bar=1 μm.

Specific PCR sequencing analysis indicated that the T-DNA was inserted at the end of the third exon, 804 bp downstream from the initiation codon of the At5g53080 locus (Fig. 1B, C). RT-PCR analysis showed that *WTG1* transcripts were absent in *wtg1-1* homologous mutants (Fig. 1D), indicating that transcription of *WTG1* was disrupted by T-DNA insertion. In addition, two other T-DNA insertion lines related to *WTG1*, SALK_071495 (*wtg1-2*) and SALK_027718 (*wtg1-3*) (Fig. 1B), were obtained from the Arabidopsis Biological Resource Center and analyzed. The T-DNAs were inserted into the beginning of the first exon and the 3′-UTR region of the At5g53080 locus in the *wtg1-2* and *wtg1-3* alleles, respectively. However, expression of *WTG1* was not disrupted in *wtg1-2* and *wtg1-3*; therefore, we chose *wtg1-1* for further analysis. To confirm that the abnormal phenotype of *wtg1-1* homozygotes was caused by disruption of At5g53080, a genetic complementation assay was performed. The complete 1692 bp CDS (excluding the stop codon) of At5g53080 was transformed into homozygous *wtg1* plants. In total, 42 T1 transgenic plants were identified; all transformants displayed normal morphology and were indistinguishable from wild-type plants (Fig. 2A). These results indicate that the *WTG1* gene is At5g53080. Moreover, the 1692 bp coding region of *WTG1* complemented the mutant phenotype.

### *WTG1* is required for chloroplast biogenesis

Chlorophyll content was measured as the leaves of *wtg1* mutants transitioned from albino to pale green to green, representing three stages of development (Fig. 2A). Consistent with the phenotypes, the chlorophyll content of the leaves of *wtg1* mutants increased as the leaves turned green, although they contained less chlorophyll than wild-type plants at each growth stage. The complemented lines possessed normal chlorophyll content (Fig. 2B).

Chloroplast biogenesis is a multistage process in which proplastids develop into fully differentiated and functional chloroplasts (Fig. 2C) (Rudowska *et al.*, 2012, Jarvis and López-Juez, 2013). Chloroplast development was examined in *wtg1* mutants using transmission electron microscopy. Albino leaves (stage 1) possessed etioplasts, whereas pale green leaves (stage 2) possessed pre-chloroplasts, and green leaves (stage 3) possessed mature chloroplasts (Fig. 2D). In contrast, mature chloroplasts were observed in the early leaves of wild-type and complemented plants (young leaves, as indicated by the arrows in Fig. 2A, were sampled) (Fig. 2D). These results indicate that chloroplast biogenesis was delayed in the *wtg1* mutants. Therefore, we conclude that *WTG1* is required for chloroplast biogenesis during the early stage of leaf development.

### WTG1 is expressed ubiquitously in Arabidopsis and localized in chloroplasts

Expression data from Genevestigator(http://www.genevestigator.com) showed that WTG1 is widely expressed in Arabidopsis (Zimmermann *et al.*, 2004). RT-PCR confirmed the ubiquitous expression pattern of *WTG1* and showed that the greatest transcript abundance was in cauline leaves, whereas low transcript abundance was measured in siliques and roots (Supplementary Fig. S2A).

To clarify the subcellular localization of WTG1, we implemented a transgenic approach to induce wild-type Arabidopsis to express a *WTG1-GFP* fusion gene under the control of the *35S* promoter. The fluorescent signal was localized within chloroplasts in homozygous transgenic plant cells (Supplementary Fig. S2B), indicating that WTG1 is a chloroplast protein.

### *WTG1* is required for plastid gene expression during early chloroplast development

**Fig. 3.**
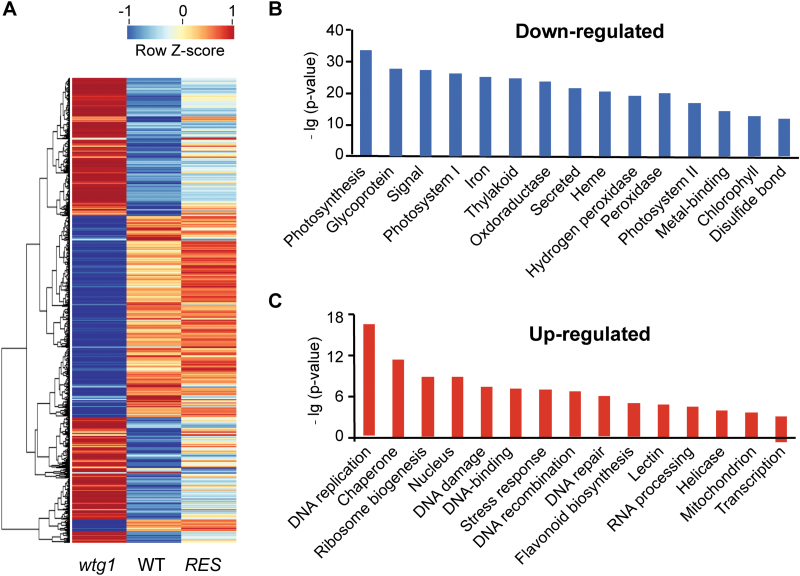
Transcriptional profiles in wild-type (WT), *wtg1* and complemented/rescued (RES) seedlings obtained by RNA-seq. (A) Heatmap for differentially expressed genes (DEGs). (B) Top 15 GO terms for DEGs down-regulated in *wtg1*. (C) Top 15 GO terms for DEGs up-regulated in *wtg1*. Genes were classiﬁed by functional categories under the ontology category of biological process.

Deficient chloroplast development is always associated with abnormal expression of plastid genes (Wang *et al.*, 2016*a*, 2016*b*, Zhang *et al.*, 2016). To analyze the effect of the *wtg1* mutation on gene expression, we determined the transcription profiles of wild-type, *wtg1*, and complemented seedlings using RNA-seq (Supplementary Fig. S3). The results showed that, in 18-day old seedlings, 5013 genes exhibited differential expression (2463 up-regulated and 2550 down-regulated) between *wtg1* and the wild type, indicating an abnormal expression pattern in *wtg1* (Fig. 3A; Supplementary Data S1 and Supplementary Fig. S3). These transcriptional abnormalities were rescued by introduction of the *WTG1* CDS into *wtg1* mutants (Fig. 3A), suggesting that *WTG1* plays a role in plastid gene expression during early chloroplast development.

To determine whether these affected genes belonged to particular gene classes, we analyzed their gene ontology (GO) classifications in the biological process category (Fig. 3B, C). The GO enrichment results showed that the down-regulated genes were significantly enriched for terms related to photosynthesis (*P*=5.3e-34) and cell communications, e.g. ‘glycoprotein’ (*P*=6.6e-29) and ‘signal’ (*P*=3.8e-28), while the up-regulated DEGs were enriched for GO terms related to DNA processing and metabolism, including ‘DNA replication’ (*P*=3.7e-17), ‘DNA damage’ (*P*=4.1e-8), ‘DNA recombination’ (*P*=1.5e-7), and ‘DNA repair’ (*P*=4.5e-7).

**Fig. 4.**
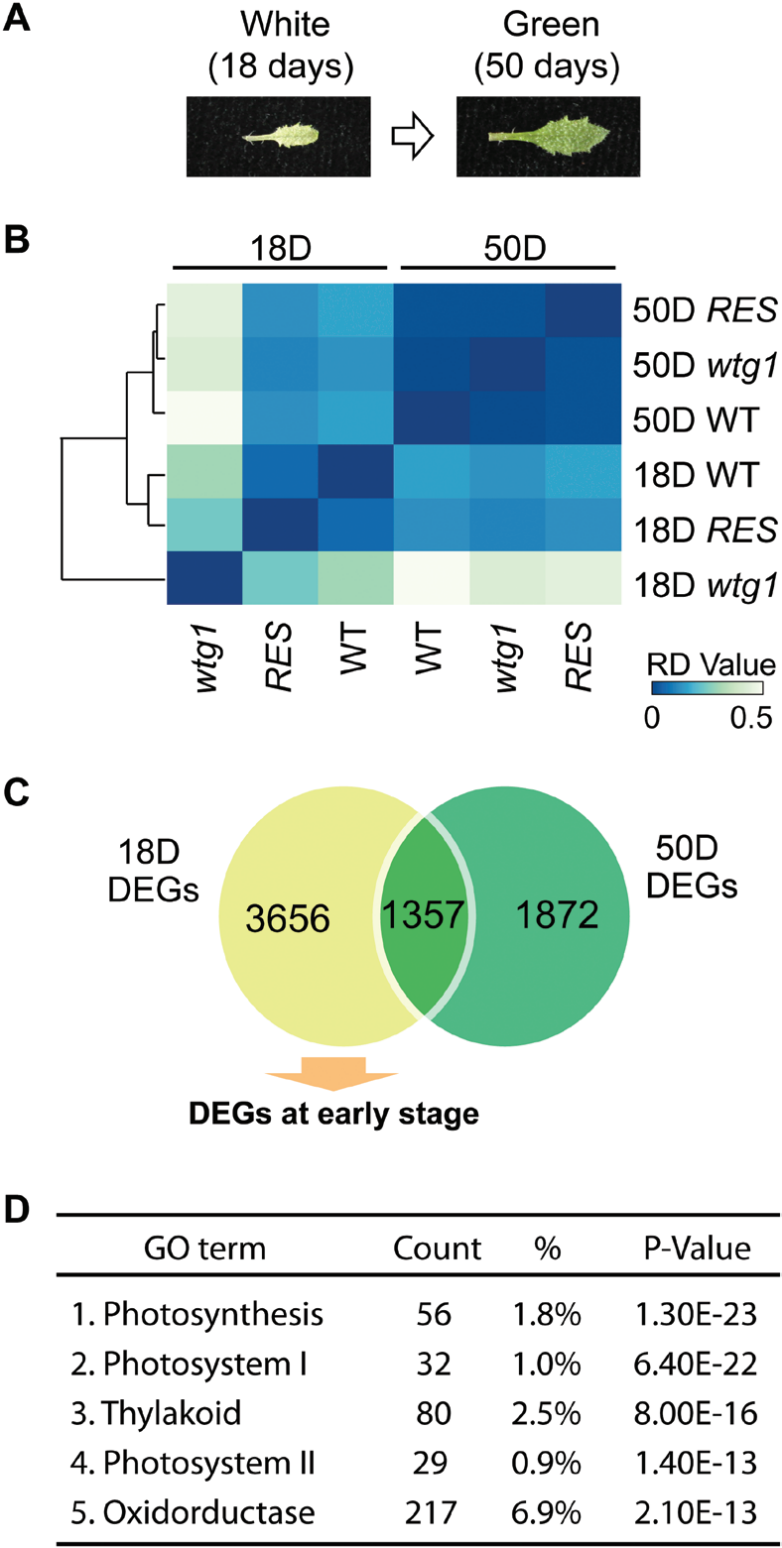
Disruption of *WTG1* causes a delayed greening phenotype and retarded developmental expression of photosystem genes. (A) *wtg1* mutants show a delayed greening phenotype. (B) Clustered heatmap visualizing the similarity relationships among samples from 18-day-old and 50-day-old *wtg1*, wild-type (WT), and complemented/rescued (RES) plants. (C) Venn diagram showing the unique and shared relationships of differentially expressed genes (DEGs) in 18-day-old and 50-day-old plants. The DEGs that existed only in 18-day-old mutant were assigned as DEGs at the early stage. (D) List of the top five gene ontology (GO) terms for the DEGs at the early stage.

To determine whether the gene expression profiles changed as *wtg1* leaves turned green, we compared the transcriptional levels in 50-day-old and 18-day-old leaves in *wtg1*, wild-type, and complemented seedlings by RNA-seq (Fig. 4A; Supplementary Fig. S3). Strikingly, the differences in gene expression profiles between 18-day-old *wtg1* and wild-type leaves were the most significant, while the expression profiles tended to be consistent between the wild type and *wtg1* in 50-day-old leaves (Fig. 4B), implying that *WTG1* mainly functions during the early stage of chloroplast development. In 18-day-old *wtg1* seedlings, there were 5013 DEGs compared with the wild-type seedlings (Fig. 4C), among which 1357 DEGs were overlapped with the DEGs in 50-day-old *wtg1* plants. Because the chloroplasts in 50-day-old green *wtg1* leaves were normal (Fig 2A, D), we hypothesized that the DEGs found only at the early stage were required for chloroplast development; thus, we performed GO enrichment analysis using the 3656 DEGs identified at the early stage (Fig. 4C, D). The results showed that the most enriched GO terms were all related to chloroplast functions, especially photosynthesis, which was consistent with our expectation (Fig. 4D).

### Expression of genes involved in photosynthesis is repressed in *wtg1*

**Fig. 5.**
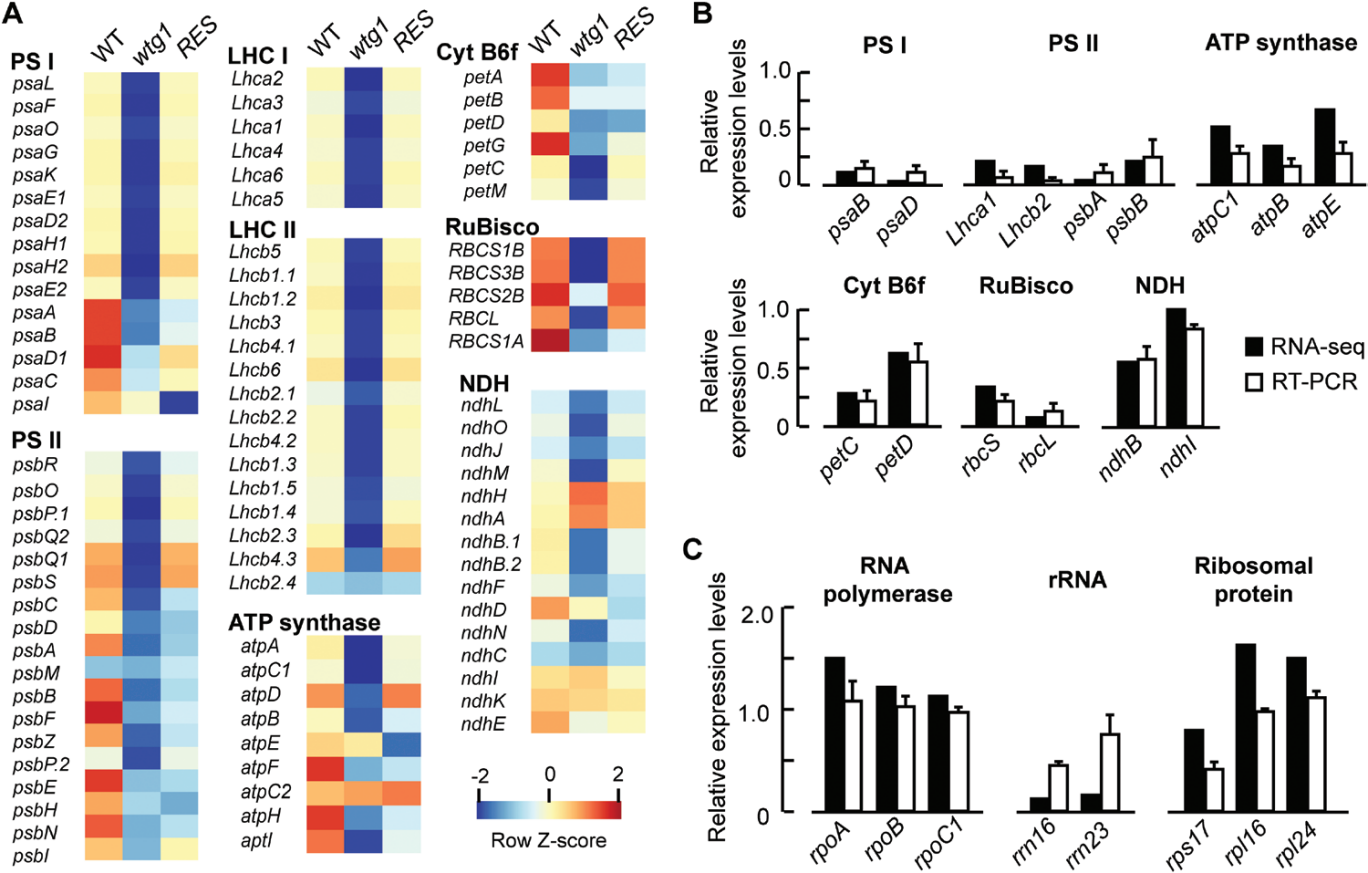
Effects of *WTG1* deficiency on the expression of chloroplast-related genes. (A) Heatmap of the transcription profiles of photosynthesis-related genes in 18-day-old seedlings of wild-type (WT), *wtg1* and complemented/rescued (RES) lines. Values were calculated as log2 ratio and colors are scaled per row, with red representing up-regulated genes and blue respresenting down-regulated genes. The heatmap was generated from http://iseq.cbi.pku.edu.cn. (B) qRT-PCR validation of the RNA-seq results. Fifteen genes were randomly selected to validate the changes in their expression levels obtained by RNA-seq (black bars) through qRT-PCR analysis (white bars). These genes belonged to different functional complexes involved in photosynthesis. (C) Expression levels of non-photosynthetic genes. Eight non-photosynthetic genes were selected to validate the changes in their expression levels obtained by RNA-seq (black bars) through qRT-PCR analysis (white bars). The expression relative to wild-type is set to 1; data are presented as mean±SD of triplicates in (B) and (C).

There are two classes of genes responsible for chloroplast biogenesis and metabolism: photosynthetic genes and non-photosynthetic housekeeping genes (Li and Chiu, 2010). To assess the impacts of *WTG1* deficiency on the expression of these genes, we compared their transcriptional profiles among 18-day-old *wtg1* mutants, wild-type, and complemented plants. As shown in Fig. 5A, many genes involved in photosynthesis were significantly repressed, including genes encoding proteins of photosystem I (PSI), photosystem II (PSII), light-harvesting complex, ATP synthase, and carbon fixation-related proteins; these results were partially veriﬁed using real-time PCR (Fig. 5B; Supplementary Fig. S4). Additionally, chlorophyll synthesis genes were down-regulated, consistent with the lower chlorophyll content in *wtg1* mutants compared with wild-type plants (Fig. 2B; Supplementary Fig. S4). Furthermore, the expression levels of most of these affected genes were rescued in the complemented lines (Fig. 5A). For non-photosynthetic housekeeping genes, the effects of *WTG1* mutation on gene expression varied. According to the RNA-seq and qRT-PCR results, the expression levels of plastid-encoded RNA polymerase genes (*rpoA*, *rpoB*, and *rpoC*1) and two ribosomal protein-encoding genes (*rpl24* and *rpl16*) in the *wtg1* mutants were almost the same as, or even higher than, those in the wild type. However, the relative expression levels of the ribosomal RNA genes *rrn16* and *rrn23* were significantly reduced in the mutant compared with the wild type. Expression of the ribosomal protein small subunit gene *rps17* was also downregulated in the *wtg1* mutants, with a transcript level of 41.61% of that in the wild type. (Fig. 5C). These results indicated that expression of the plastid genes was affected by the *wtg1* mutation.

### The *WTG1* gene encodes a TPR protein

**Fig. 6.**
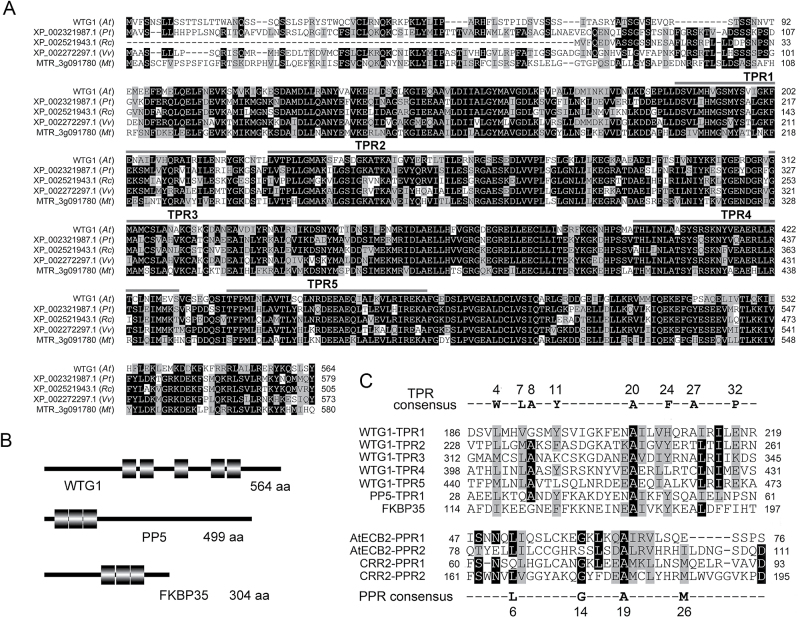
WTG1 belongs to the TPR protein family. (A) Sequence alignment of WTG1 from *Arabidopsis thaliana* (*At*) and its homologs from *Populus trichocarpa* (*Pt*), *Ricinus communis* (*Rc*), *Vitis vinifera* (*Vv*), and *Medicago truncatula* (*Mt*). The sequences were aligned using ClustalW (Thompson *et al.*, 1994) with the conserved residue shading mode. The TPR motifs are indicated at the top of each sequence. (B) Schematic diagrams of WTG1 and two other typical TPR proteins. Black rectangles represent TRP motifs. (C) Alignments and comparisons of TPR and PPR motifs. The upper sequence alignment consists of five TPR motifs in WTG1 and the first TPR motifs of two typical TPR proteins, PP5 and FKBP35. The reported TPR consensus is aligned at the top. The lower sequence alignment consists of two PPR motifs of the PPR proteins AtECB and CRR2. The reported PPR consensus is aligned at the bottom. The alignment was performed using ClustalW with the conserved residue shading mode.

BLAST searches demonstrated that *WTG1* is a single-copy nuclear gene that is conserved in flowering plants and encodes a putative polypeptide of 564 amino acids with a calculated molecular mass of 63.1 kDa (Fig. 6A). Domain analysis by PROSITE and SMART revealed that WTG1 possesses five TPR motifs (Fig. 6B), which meets the TPR protein definition criterion (i.e. containing 3–16 TPR motifs) (Blatch and Lässle, 1999).

The sequence of WTG1 was compared with those of well-characterized TPR proteins: human Ser/Thr phosphatase PP5 and plasmodium FKBP35 (Das *et al.*, 1998, Alag *et al.*, 2009). Using Bioedit software, we found that the five TPR coding sequences in WTG1 shared 60% similarity with the first TPR coding sequences of PP5 and FKBP35 (Fig. 6C). In particular, all five WTG1 TPR motifs contained the conserved residues typical of the TPR consensus sequence at positions 4 (W/L/F), 7 (L/I/M), 8 (G/A/S), 11 (Y/L/F), 20 (A/S/E), 24 (F/Y/L), 27 (A/S/L), and 32 (P/K/E) (Lamb *et al.*, 1995, D’Andrea and Regan, 2003). The TPR motifs of WTG1 were then compared with the motif sequences of Arabidopsis PPR proteins AtECB2 and CRR2 by using the consensus sequence at positions 6 (L), 14 (G), 19 (A), and 26 (M) (Hashimoto *et al.*, 2003, Yu *et al.*, 2009) for comparisons (Fig. 6C), but revealed no similarities. These results indicate that WTG1 is a TPR protein rather than a PPR protein.

### Disruption of *WTG1* has an effect on RNA editing of *petL* and *ndhG*

**Fig. 7.**
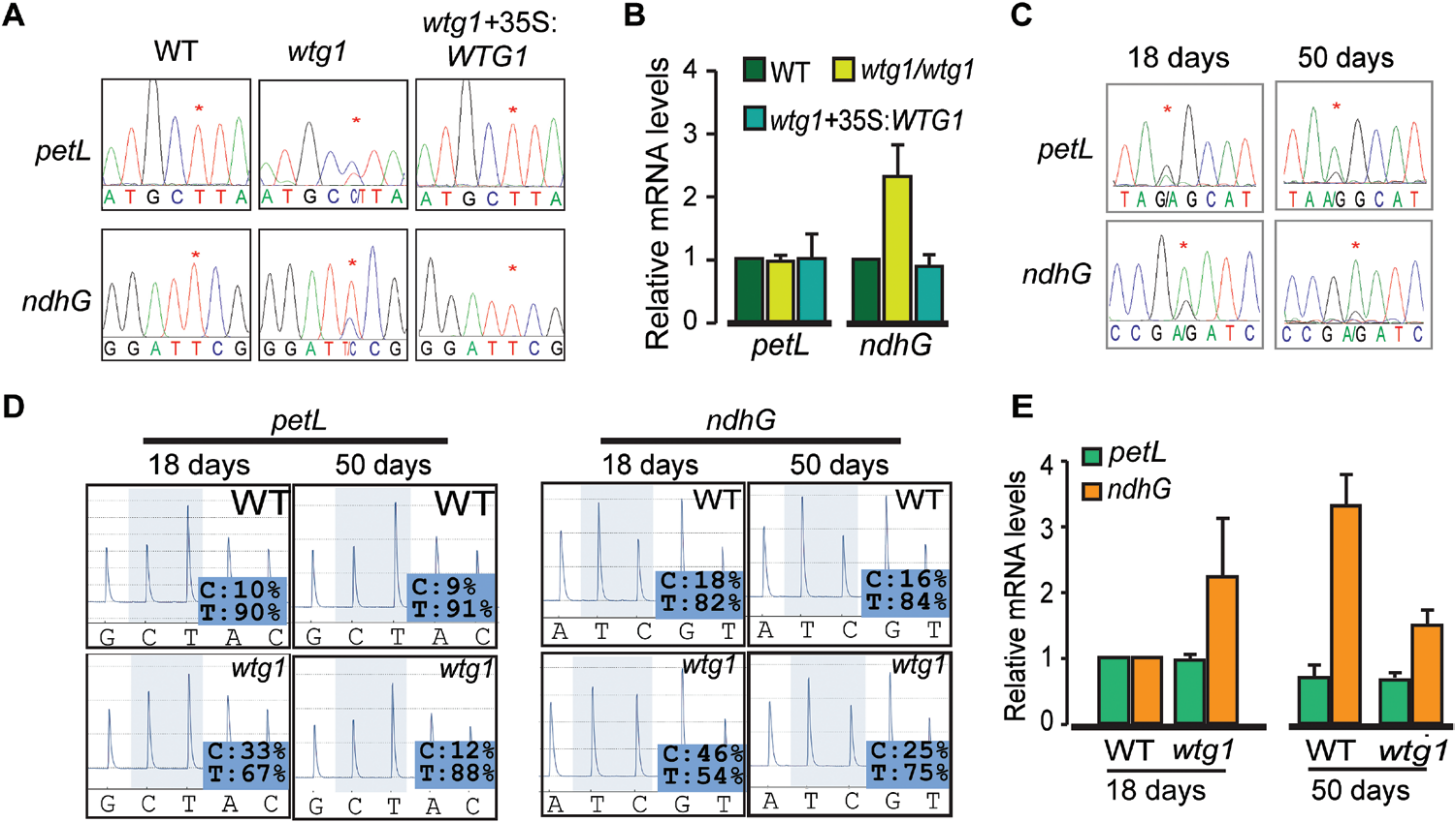
*WTG1* is required for RNA editing at *petL-5* and *ndhG-50* sites in plastids. (A) Loss of *WTG1* disrupts editing of plastid *petL-5* and *ndhG-50* sites in 18-day-old seedlings as demonstrated by bulk sequencing. Asterisks indicate editable Cs. (B) Relative mRNA abundance of *petL* and *ndhG* in 18-day-old seedlings of wild-type (WT), *wtg1*, and *wtg1* complemented with *Pro35S:WTG1*. Data are presented as mean±SD of triplicates. (C) Defects in editing of *petL-5* and *ndhG-50* sites are restored as leaves turn green. Asterisks indicate editable Cs. (D) Validation of *petL-5* and *ndhG-50* editing extent in WT and *wtg1* mutant plants by pyrosequencing. (E) Relative mRNA abundance of *petL* and *ndhG* in WT and *wtg1* plants at different growth stages. Data are presented as mean±SD of triplicates.

The albino phenotype with chloroplast biogenesis deficiency is reminiscent of some mutants with defective plastid RNA editing (Chateigner-Boutin *et al.*, 2008, Yu *et al.*, 2009, Zhou *et al.*, 2009, Takenaka *et al.*, 2012, Sun *et al.*, 2015, Yap *et al.*, 2015, Zhang *et al.*, 2015). We therefore compared the states of chloroplast RNA editing in 18-day-old wild-type plants, *wtg1* mutants, and the complemented transgenic line by bulk sequencing. Of the 34 plastid RNA editing sites reported previously (Tillich *et al.*, 2005, Chateigner-Boutin and Small, 2007, Bentolila *et al.*, 2012, Ruwe *et al.*, 2013), 32 sites in the *wtg1* mutants were edited in the same manner as those of the wild-type plants; however, the other two editing sites, in *petL-5* and *ndhG-50*, exhibited remarkable differences (Fig. 7A; Supplementary Table S2). The *petL-5* editing rate in the *wtg1* mutants was approximately 40%, which was remarkably lower than that of the wild-type plants (nearly 100%). Similarly, the editing rate of the *ndhG-50* site in the *wtg1* mutants was approximately 60%, which was notably lower than that of the wild-type plants (nearly 100%). It is notable that the editing rates of both sites within the complemented transgenic line were identical to those of the wild-type plants (both nearly 100% edited). The transcript abundance of *petL* was unchanged in the *wtg1* mutants, whereas that of *ndhG-50* was increased for unknown reasons (Fig. 7B). These results indicate that WTG1 protein is required for full editing of *petL-5* and *ndhG-50* transcripts in early leaves.

To detect whether these editing deficiencies could recover as albino leaves turned green, we sampled pale green (18-day-old) and green (50-day-old) leaves of *wtg1* mutant plants and examined the extent of editing in RNA transcripts from these leaves. Bulk sequencing showed that the editing rates of *petL-5* and *ndhG-50* were mostly restored during leaf growth and greening (Fig. 7C). The editing rates of *petL-5* and *ndhG-50* were approximately 45% and 70%, respectively, in pale green leaves, but reached approximately 80% and 90%, respectively, in green leaves. Compared with bulk sequencing, pyrosequencing is a more sensitive and quantitative method of assessing RNA editing. Therefore, we reassessed the extent of RNA editing using pyrosequencing (Fig. 7D). The editing rate of *petL-5* in pale green leaves was 67%, but this rate increased to 88% in green leaves; this editing rate was nearly equal to that of the wild-type plants (approximately 90% as determined with this method). At the *ndhG-50* site in *wtg1* mutants, the editing rate was 54% in pale green leaves and 75% in green leaves, while the editing rate of wild-type plants was determined to be approximately 83% using pyrosequencing. Moreover, we detected a decrease in *ndhG* and *petL* RNA abundance in the 50-day-old leaves compared with the 18-day-old leaves of *wtg1* (Fig. 7E), implying that the increased editing rate in green leaves may be caused by the decrease of transcript abundance.

### WTG1 may affect RNA editing through interactions with MORFs

**Fig. 8.**
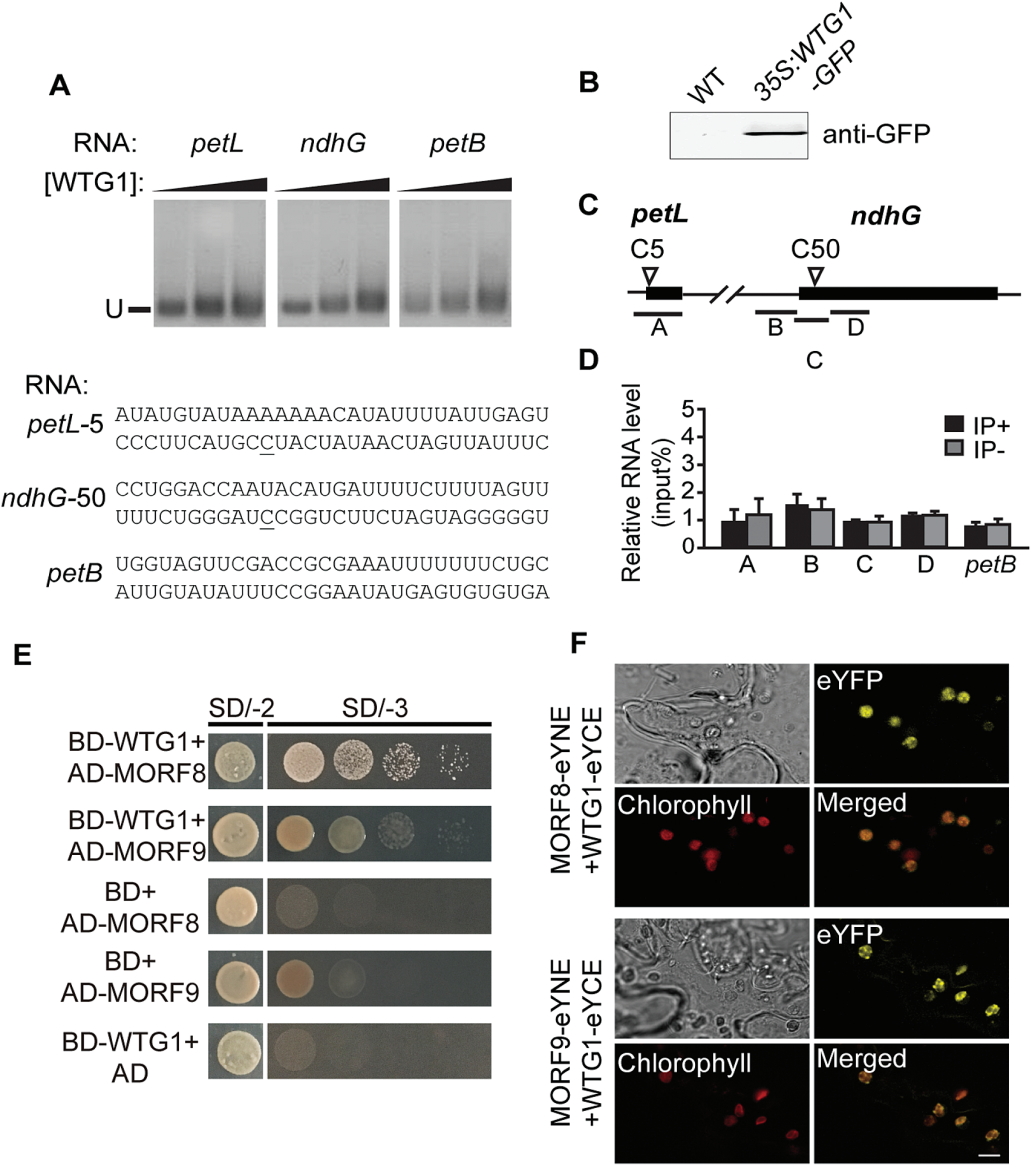
WTG1 has no association with *petL* and *ndhG* transcripts but interacts directly with MORF8 and MORF9. (A) REMSA showing no binding of WTG1 to the *petL* or *ndhG* transcript. The assays were performed with WTG1 protein at the indicated concentrations and the biotin-labeled RNAs shown below (edited site underlined). The *petB* sequence was not edited and served as a negative control. U, unbound RNA. (B) Validation of WTG1-GPF protein expression. Total protein was extracted from 18-day-old wild-type (WT) and *35S:WTG1-GFP* transgenic plants, after which immunoblot analysis was performed with anti-GFP antibodies. (C) Diagram of *petL* and *ndhG* genes analyzed in the RIP assay. Regions analyzed by PCR are underlined. (D) RNA immunoprecipitation followed by a qRT-PCR assay using *35S:WTG1-GFP* plants and anti-GFP antibodies. IP+, anti-GFP immunoprecipitation; IP-, mock immunoprecipitation. Data are presented as mean±SD of triplicates. (E) Yeast two-hybrid assay. AD, GAL4 activation domain; BD, GAL4 DNA binding domain. SD/-2 and SD/-3 indicate SD/-Trp-Leu and SD/-Trp-Leu-His dropout plates, respectively. Yeast colonies grown on SD/-3 plates indicate interaction between proteins. (F) Bimolecular fluorescence complementation assay showing the interactions between WTG1 and MORF8 or MORF9, which lead to the production of YFP fluorescence in chloroplasts. Chlorophyll autofluorescence is shown in red. Bar=5 μm.

We presumed that WTG1 may participate in RNA editing through protein–protein interactions, as is typical for TPR proteins (D’Andrea and Regan, 2003), rather than by recognizing the *cis*-element 5′-adjacent to the target editable Cs, as is typical for PPR proteins (Chaudhuri and Maliga, 1996, Okuda *et al.*, 2006, Hayes and Hanson, 2007). To assess this possibility, we first performed RNA binding assays with purified WTG1 protein. Gel mobility-shift assays were performed with WTG1 and synthetic RNAs of WTG1-related editing sites *petL-5* and *ndhG-50*. A *petB* 5′-UTR lacking editing sites was used as the negative control (Sun *et al.*, 2013). WTG1 exhibited no affinity for any of the three tested RNAs, showing that WTG1 does not bind to the two putative *cis*-elements of the *petL-5* and *ndhG-50* editing sites (Fig. 8A). To verify this result, RNA immunoprecipitation assays were performed (Fig. 8B–D). There was no pronounced enrichment of *petL-5* and *ndhG-50* in comparison with the *petB* 5′-UTR control in RNA samples isolated from *35S:WTG1-GFP* transgenic plants, indicating that WTG1 does not specifically associate with these target sequences in the transcripts.

Next, we employed a series of yeast two-hybrid assays to assess whether WTG1 interacts with editing factors at the *petL-5* and *ndhG-50* sites (Supplementary Table S3). These experiments were based on our presumption that WTG1 may participate in RNA editing through protein–protein interactions. WTG1 interacted with MORF8 and MORF9, but not with other factors known to be relevant to *petL-5* and/or *ndhG-50* editing, including OTP82, MORF2, ORRM1, OZ1, and PPO1 (Fig. 8E). Direct interaction between WTG1 and MORF8 and MORF9 was verified using a BiFC assay. Co-expression of the C-terminal YFP fusion of WTG1 (WTG1-eYCE) and the N-terminal YFP fusion of MORF8 (MORF8-eYNE) or MORF9 (MORF9-eYNE) reconstituted a functional YFP in chloroplasts (Fig. 8F; Supplementary Fig. S5). These results provide evidence that WTG1 may affect RNA editing through physical interactions with MORF proteins in chloroplasts.

## Discussion

### WTG1 is a TPR protein required for early chloroplast development

Disrupted chloroplast development usually results in abnormal leaf coloration, which severely impacts the biomass or survival of plants. Many mutants with delayed greening characteristics have been described. The *dg1* mutant has very pale young leaves but greens gradually, eventually appearing similar to wild-type plants (Chi *et al.*, 2008). In *sg1* mutants, the newly formed leaves are initially albino but the wild-type phenotype is restored after 3 weeks (Hu *et al.*, 2014). Mutation of the purine biosynthetic enzyme ATase2 of Arabidopsis results in chlorotic young leaves, which recover to be green upon maturity (Yang *et al.*, 2015). In another recently identified mutant, *dg238*, the young leaves exhibit a chlorotic phenotype but this lessens as the plant develops (Wang *et al.*, 2016*a*). In this study, we reported the characterization of a new mutant, *wtg1*, which also has a pronounced delay in greening and exhibits dwarf and serration phenotypes as well. Molecular cloning and complementation assays revealed that the phenotype of *wtg1* was controlled by a recessive gene that encodes a TPR-containing protein.

The most interesting phenotype of *wtg1* is the retarded greening of both its cotyledons and true leaves. The young leaves of *wtg1* mutants were initially albino, and then gradually greened during development, indicating that WTG1 plays an important role in the early stages of chloroplast development. Like *wtg1*, all the above-mentioned mutants with delayed greening characteristics exhibit a severe chlorotic phenotype with defective chloroplasts only at the early stage of plant development; once fully grown, the chloroplasts become normal, making the plants photoautotrophic. There are two possible explanations for this phenomenon. One is that other proteins may show functional redundancy. That is, some homologous proteins or factors with similar functions may exist and partly compensate for the mutant proteins during later stages of development (Ishizaki *et al.*, 2005, Chi *et al.*, 2008). However, when we performed protein alignments we found no other proteins in Arabidopsis with homology to WTG1. The other possibility is that chloroplast development is an integrated outcome that depends on the balance of plastid protein accumulation and degradation. According to a previously hypothesized threshold model (Yu *et al.*, 2005, 2008, Wang *et al.*, 2016*b*), the products of plastid genes may accumulate at a faster rate than they are than degraded in the *wtg1* mutant during plant growth; when the concentrations of certain proteins exceed a threshold, normal chloroplasts are produced, and the leaves turn green.

### WTG1 plays an important role in the regulation of chloroplast gene expression

Chloroplast development requires the coordinated expression of genes encoded by both the nuclear and plastid genomes. Disruption of *WTG1*, a nuclear gene, dramatically reduced the expression levels of chloroplast-related genes (Fig. 5), indicating an important role for WTG1 in regulating chloroplast gene expression. In plastids, gene transcription depends on two types of RNA polymerases: the nuclear-encoded plastid RNA polymerase (NEP) and the plastid-encoded plastid RNA polymerase (PEP) (Maliga *et al.*, 1988, Hajdukiewicz *et al.*, 1997, Hedtke *et al.*, 1997). NEP mainly transcribes non-photosynthetic housekeeping genes, while PEP specifically transcribes the photosynthetic genes. According to our RNA-seq results, the expression of most NEP- and PEP-transcribed genes was altered significantly in the *wtg1* mutant (Supplementary Fig. S4), implying a distinct role of WTG1 in regulating gene expression compared with factors specifically involved in PEP transcription, such as the DG1, ATase2, and DG238 proteins (Chi *et al.*, 2008, Yang *et al.*, 2015, Wang *et al.*, 2016*a*). In addition, we observed reduced expression of most nuclear-encoded chloroplast genes and chlorophyll biosynthesis genes (Fig. 5; Supplementary Fig. S4). It has been reported that the expression of a set of nuclear genes that encode chloroplast-localized proteins is controlled by signaling from the chloroplast via a process called retrograde signaling (Oelmuller, 1989, Surpin *et al.*, 2002). We examined the expression levels of GUN1, GUN2 (Susek *et al.*, 1993), EXECUTER1 and EXECUTER2 (Kim and Apel, 2013), which were proven to be involved in retrograde signaling, in *wtg1* mutants. Although the expression level of *EXECUTER2* was decreased, the transcript abundance of three other genes in *wtg1* was equivalent to that in the wild type (Supplementary Fig. S4), suggesting that WTG1 is unlikely to be an upstream regulator in the retrograde signaling pathway. However, WTG1 may participate in the retrograde signaling process through protein–protein interactions or other unknown mechanisms. Further studies are required to clarify the mechanisms by which WTG1 regulates chloroplast development.

### WTG1 may be involved in RNA editing of *petL* and *ndhG* transcripts

The extent of RNA editing can vary with RNA abundance. This can be observed in the correlation between transcript and editing levels. Reported examples include the mitochondrial gene *nad3* (*NADH-dehydrogenase subunit 3*) in a *Petunia* hybrid (Lu and Hanson, 1992) and plastid genes in *Nicotiana tabacum* and *Zea mays* (Chateigner-Boutin and Hanson, 2002, Peeters and Hanson, 2002). However, the direct factor that affects RNA editing is the efficiency of the editosome, the functional editing protein complex composed of PPRs, MORFs, and other components. Experimental evidence has verified that when a component is deficient, the editing of one or several sites is impaired (Yu *et al.*, 2005, Mach, 2009, Bentolila *et al.*, 2012, Takenaka *et al.*, 2012, Sun *et al.*, 2013, 2015, Zhang *et al.*, 2014, Shi *et al.*, 2016*b*).

Our results showed that the expression profiles of plastid-encoded genes in *wtg1* were largely altered compared with the wild type (Fig. 5). As described above, we cannot exclude the possible influence of RNA editing in *wtg1* plants on the changes in RNA abundance. However, after careful analysis of our results, we believe that the influence of *petL* and *ndhG* RNA editing on RNA abundance in *wtg1* is minor; that is, WTG1 is likely to participate in RNA editing. This is because, among the genes with greatly altered transcript abundances, such as *accD*, *psbF*, and *ndhB* (Fig. 5; Supplementary Fig. S4), only two sites in *petL* and *ndhG* showed a deficiency in RNA editing (Supplementary Table S2). In fact, the abundance of *petL* transcripts remained unchanged in *wtg1* plants (Fig. 7B). More importantly, our results confirmed that WTG1 interacts with the known editing factors MORF8 and MORF9 (Fig. 8E, F). These results provide evidence that WTG1 affected RNA editing and suggest the involvement of WTG1 in the editing process. Given that WTG1 does not bind RNA (Fig. 8A–D), consistent with the notion that the TPR domain mainly mediates protein–protein interactions (Das *et al.*, 1998, Blatch and Lässle, 1999, D’Andrea and Regan, 2003, Zeytuni and Zarivach, 2012), we propose that the involvement of WTG1 in RNA editing would be a procedure that stabilizes the editosome through interaction with MORFs. If this is true, WTG1 would add another level of complexity to the plant editosome. This would shed light on the mechanism of plant RNA editing because, to our knowledge, the involvement of a TPR protein in RNA editing has not been suggested before.

## Supplementary data

Supplementary data are available at *JXB* online.

Fig. S1. Loss of *WTG1* reduces the yield trait of Arabidopsis.

Fig. S2. WTG1 is expressed ubiquitously in Arabidopsis and targeted to chloroplasts.

Fig. S3. RNA-seq analysis of wild-type and *wtg1* leaves.

Fig. S4. Transcript analysis of chloroplast-associated genes in 18-day-old *wtg1* by RNA-seq.

Fig. S5. Controls for the bimolecular fluorescence complementation assay.

Table S1. List of primers used in this study.

Table S2. Plastid RNA editing sites affected in the *wtg1* mutant plants.

Table S3. *Trans*-factors involved in *petL-5* and/or *ndhG-50* editing.

Data S1. List of genes that are differentially expressed in wild-type and *wtg1* plants.

## Supplementary Material

supplementary_figures_S1_S5_tables_S1_S3

supplementary_dataset_S1
