# Supplementary material for: A novel tetratricopeptide repeat protein, WHITE TO GREEN1, is required for early chloroplast development and affects RNA editing in chloroplasts
Source: J Exp Bot. 2017 Nov 11;68(21-22):5829–43. doi: 10.1093/jxb/erx383 (PMC5854136; doi:10.1093/jxb/erx383)
Supplement: supplementary_figures_S1_S5_tables_S1_S3 [file erx383_suppl_supplementary_figures_s1_s5_tables_s1_s3.pdf]

Supplementary data

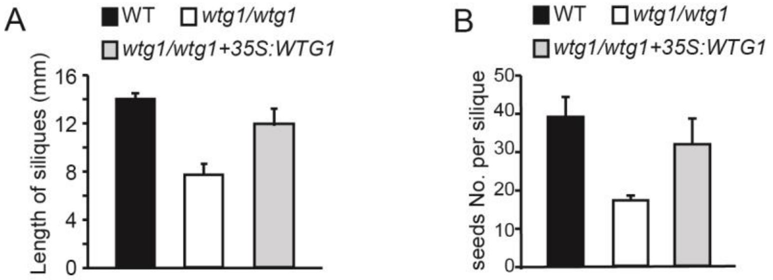

Figure S1. Loss of *WTG1* reduces the yield trait of Arabidopsis. (A) The length of siliques from wild-type, *wtg1*, and complementary lines. (B) Seed setting ability of wild type, *wtg1*, and complementary lines. Data are presented as mean ± SD of triplicates.

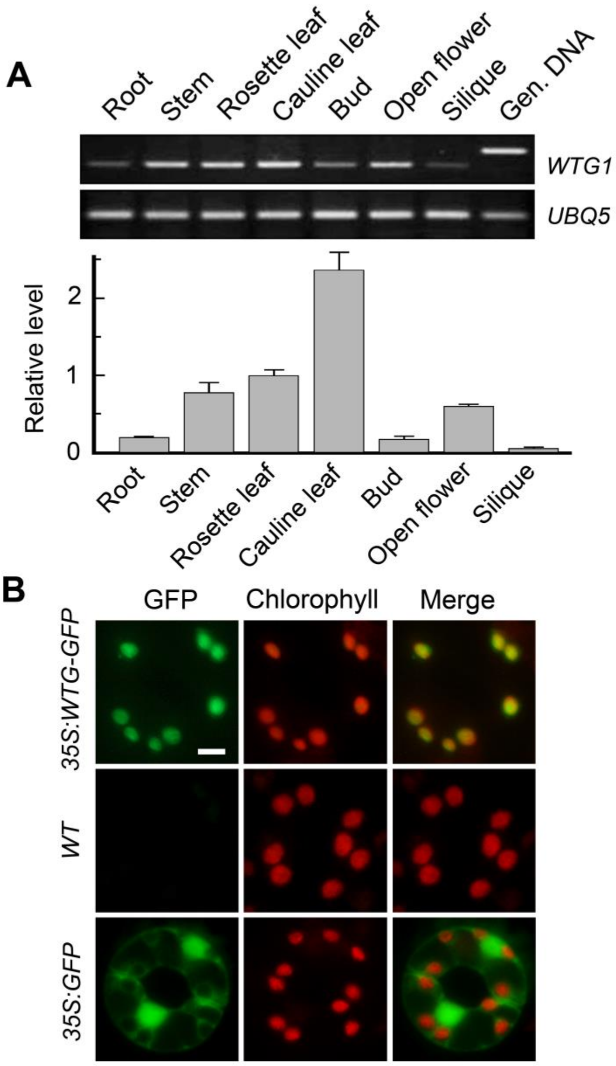

Figure S2. WTG1 is expressed ubiquitously in *Arabidopsis* and targeted to chloroplasts. (A) Reverse-transcriptase PCR (upper) and real-time PCR (bottom) assays of *WTG1*. *UBQ5* served as the loading control; data are presented as mean ± SD of triplicates. (B) Subcellular localization of WTG1-GFP in transformed mesophyll cells. Chlorophyll autofluorescence is shown in red. The image appears yellow where the signals from GFP and chloroplasts overlap. Bars = 10 μm.

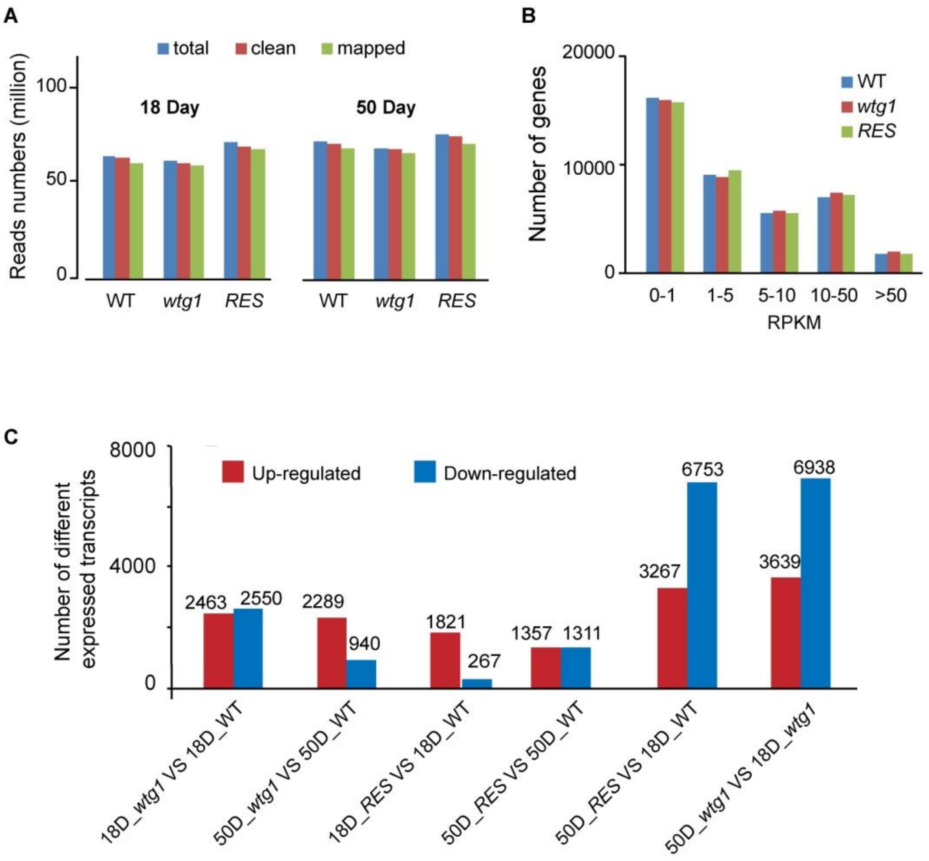

Figure S3. RNA-seq analysis of wild-type and wtg1 leaves (A) Read numbers of sample sequences. (B) Frequencies of detected genes sorted according to their expression levels. (C) Number of differentially expressed transcripts.

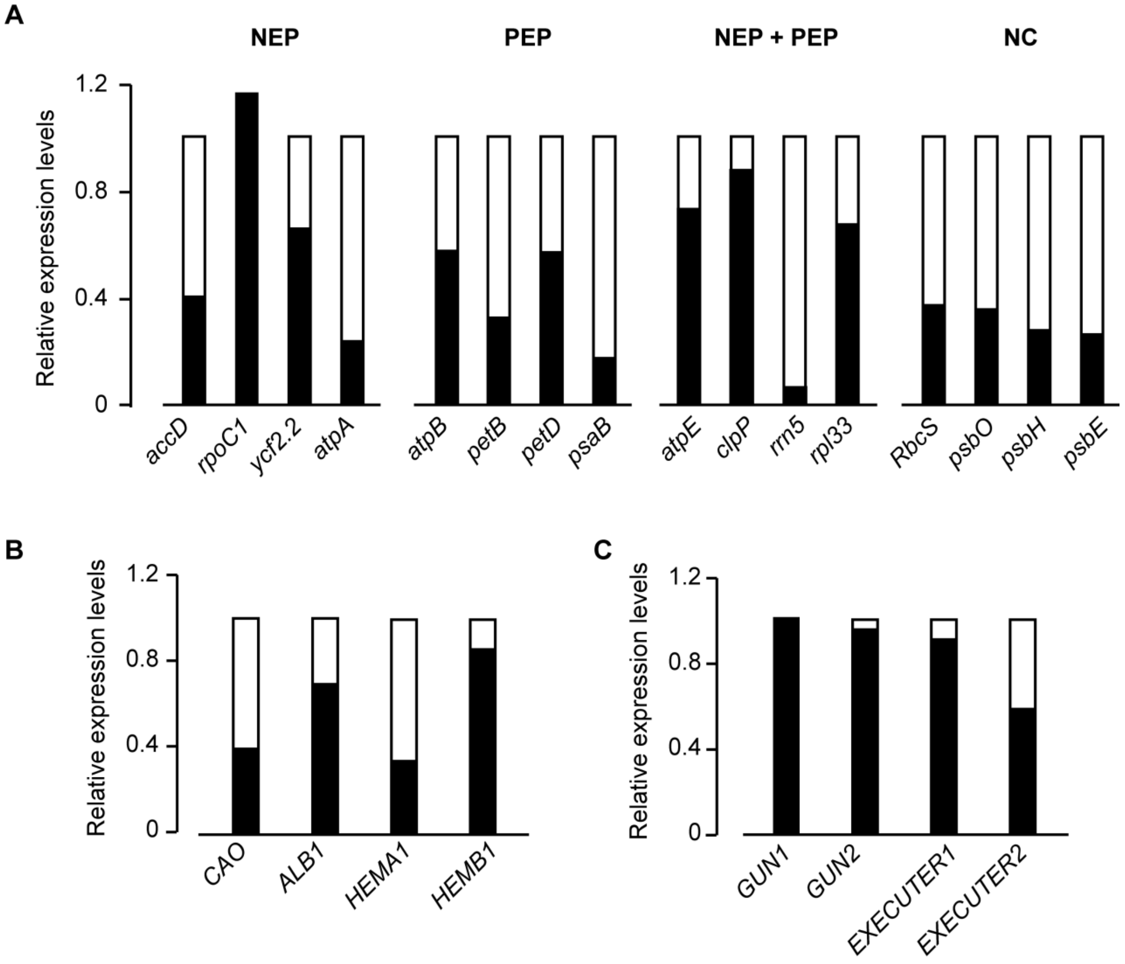

Figure S4. **Transcript analysis of chloroplast-associated genes in 18-day-old *wtg1* by RNA-seq.** The relative expression levels are represented by the RNA-seq fold change values with *wtg1* in black bars and WT in white bars (fold change value = 1). (A) The expression levels of plastid genes transcribed by nuclear-encoded polymerase (NEP), plastid-encoded polymerase (PEP), both nuclear-encoded and plastid-encoded polymerase (NEP + PEP) and nuclear-encoded chloroplast genes (NC). (B) The expression levels of chlorophyll biosynthesis genes. (C) The expression levels of genes involved in retrograde signaling.

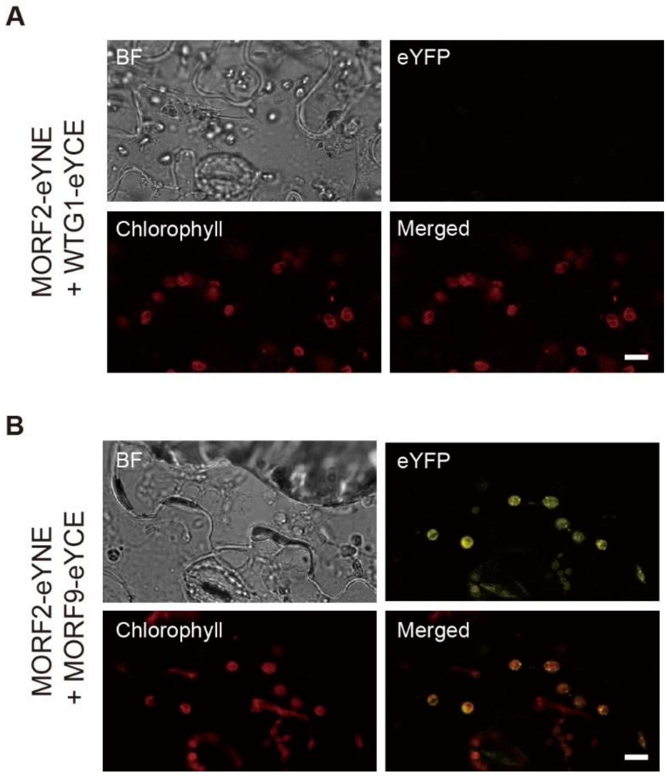

Figure S5. **Controls for the Bimolecular fluorescence complementation assay.** (A) The negative control showed no interactions between WTG1 and MORF2. (B) The interaction between MORF2 and MORF9 was used as the positive control (Takenaka *et al.*, 2012). Chlorophyll autofluorescence is shown in red in (A) and (B). Bars = 5 μm.

Table S1 List of primers used in this study.

Primer name	Sequences (5′-3′)	Purpose
53080cds_L1	CGCGGATCCATGGTGTTCTCTAACTCTC	
53080link_R1	GACTAGACCTCCGCCGTAGCTAAGACTCTGCTTG	
linkGFP_L1	GCCGGCGGAGGTCTAGTCATGGTGAGCAAGGGCGAG	
linkGFP_R1	CGCGTCGACTTACTTGTACAGCTCGTCC	
BamHI-53080-F	CACGGATCC ATGGTGTTCTCTAACTCTCTCC	
53080- EcoR1-R	CCCGAATTC CTAGTAGCTAAGACTCTGCTTG	
L1	TGGCAGTGTGTTTGTTTACG	
R1	TTGAGCAGAAGTTTACCGAG	
L2	TGTCCTTGTGCCTTCTCG	
R2	GAAGGAAGTTGGAAATGGAT	
071495-LP	CCTCAAAAGACGACAAACCTG	
071495-RP	GAAGCCGTTATTGAGGAGGAG	
006120-LP	TGCTTCAGCAGCTTTACCTTC	
006120-RP	TTTCAGGGCAAATCAATCAAG	
015164-LP	TCAGGATATAGCTGGTGGTGG	RT-PCR
015164-RP	GAGGTGGAAAAACTCTGGCTC	
LBa1	TGGTTCACGTAGTGGGCCATCG	RT-PCR
UBQ5F	AAGGTTCAGCGTTTGAGGAAGG	
UBQ5R	TCTTTCTGGTAAACGTAGGTGAGTC	RT-PCR
Y2H-EcoRI-53080F	GCGAATTCATGGTGTTCTCTAACTCTCT	
Y2H-53080-SalIR	GCGTCGACCTAGTAGCTAAGACTCTGCT	
Y2H-EcoRI-otp82F	GCGAATTCATGATGCTCT CGTGTTCTCC	
Y2H-otp82-BamHIR	GCGGATCCCTACCAGTAGTCATTGCAGG	
Y2H-EcoRI-morf2F	GCGAATTCATGGCTTTGC CTTTGTCTGG	
Y2H-morf2-SacIR	GCGAGCTCTCATCTTGTGTTTTCTCTGC	
Y2H-EcoRI-morf8F	GCGAATTCATGGCGACGC ATACCATTTC	
Y2H-morf8-BamHIR	GCGGATCCTTAACCCTGGTAGGGGTTGC	
Y2H-EcoRI-morf9F	GC GAATTCATGGCTTCCT TCACAACAAC	
Y2H-morf9-BamHIR	GCGGATCCTTAAGAGGAATCAGAGGCTG	
Y2H-EcoRI-orrm1F	GCGAATTCATGGAAGCTC TTATTGCTTC	
Y2H-orrm1-BamHIR	GCGGATCCCTAGAGCCCGAAACTTGGTT	
Y2H-NdeI-OZ1F	GGCATATGATGAACAACT CCACCAGACT	
Y2H-OZ1-BamHIR	GCGGATCCTCATTTATCTCCTTTACCAG	
Y2H-EcoRI-ppo1-F	GCGAATTCATGGAGTTAT CTCTTCTCCG	
Y2H-ppo1-BamHI-R	GCGGATCCTTACTTGTAAGCGTACCGTG	
BiFC-BamHI-53080F	CGCCACTAGTGGATCCATGGTGTTCTCTAACTCTCT	

BiFC-53080-SalIR	TACCCTCGAGGTCGACGTAGCTAAGACTCTGCTTGT
BiFC-BamHI-morf2F	CGCCACTAGTATGGCTTTGC CTTTGTCTGG
BiFC-morf2-SalIR	TACCCTCGAGTCATCTTGTGTTTTCTCTGC
BiFC-BamHI-morf8F	CGCCACTAGTGGATCCATGGCGACGCATACCATTTC
BiFC-morf8-SalIR	TACCCTCGAGGTCGACACCCTGGTAGGGGTTGCCAC
BiFC-BamHI-morf9F	CGCCACTAGTGGATCCATGGCTTCCTTCACAACAAC
BiFC-morf9-SalIR	TACCCTCGAG GTCGACAGAGGAATCAGAGGCTGCTG
petL-pyro-F	AACATATTTTATTGAGTCCCTTC
petL-pyro-R (biotinylated)	TTGCTTAGACCAATAAACAGAAC
ndhG-pyro-F	GATTTTCTTTTAGTTTTTCTGGG
ndhG-pyro-R biotinylated)	TACAAACAAGAACAAATCCTAAG
petLq-F	ATGC**C**TACTATAACTAGTTATTTC
petLq-R	TCAAATAAGTCGTATTTTGCTTAG
ndhGq-F	TGGTACGGGGTTATTTGGACTAC
ndhGq-R	CACCTATCAAAGCAACTAAAAGAATT
18srRNA-F	AAACGGCTACCACATCCAAG
18srRNA-R	ACTCGAAAGAGCCCGGTATT
psaB-F	GGACCCCACTACTCGTCGTA
psaB-R	ATTGCTAATTGCCCGAAATG
psaD-F	GATCTTCAACTCCGCCATAA
psaD-R	ATCCCACGGGAGCTTCTTTC
Lhca1-F	CAGGAATGGGCAGCACTA
Lhca1-R	GCAATGGCTAAGAACTCAAT
Lhcb2-F	CTCCCCAAAGCATCTGGTAT
Lhcb2-R	CCCATCTGCTGTGGATTACTT
psbA-F	CCTATGGGGTCGCTTCTGTA
psbA-R	GAGCAGCAATGAATGCGATA
psbB-F	CGTGCGACTTTGAAATCTGA
psbB-R	TAGCACCATGCCAAATGTGT
atpC1-F	CGTGGATTATGTGGTGGA
atpC1-R	GCTTCTTTAGCCGTAGGT
atpB-F	GAGCTCGTATGAGAGTTGGT
atpB-R	ACCCAATAAGGCGGATACCT
atpE-F	TCCACAAGAAGCTCAGCAAA
atpE-R	GTGTCCGAGCTCGTCTGAG
petC-F	CAAGTGGGTTGTTTGTGA

petC-R	AAGCATGTAGCCAGTAGGT	
petD-F	TCCTTTTGCAACTCCTTTGG	qPCR
petD-R	CCGCTGGTACTGAAACCATT	
rbcS-F	CTTCCTGACCTTACCGATTC	
rbcS-R	CGGGTGAGTTACCGTGCT	
rbcL-F	GTGTTGGGTTCAAAGCTGGT	
rbcL-R	CATCGGTCCACACAGTTGTC	
ndhB-F	GGCCTATATTTCTTGGTTTCAATAG	
ndhB-R	AATCGGGTTCATTGATATTCCTG	
ndhI-F	GTCAACAAACCCTACGAG	
ndhI-R	ACATACTTCACAAGCAAT	
rpoA-F	CAAGCCGACA CAATAGGCAT	
rpoA-R	AGCGCGTTGCGCGTTCCATA	
rpoB-F	GCAGGTTAGAATTAGAGATTGATA	
rpoB-R	GGGTAGCAAACATTCTCTAGAAT	
rpoC1-F	GCCTAGTATACTGCGATTTTTC	
rpoC1-R	CTCGATTTCGAAATATATCGAAAC	
rrn16-F	CGGTATCTGGGGAATAAGCA	
rrn16-R	GATTTGACGGCGGACTTAAA	
rrn23-F	GGGCGACTGTTTACCAAAAA	
rrn23-R	TTACCCGACAAGGAATTTCG	
rpl24-F	TTGTCGGTGCTACTTTGG	
rpl24-R	CTCGTCCTTGGTCTTCTTGATT	
rpl16-F	AACACTTGAACCCGCTTGG	
rpl16-R	GCGTTTCCGCAGGTCTTA	
rps17-F	CCCGAACCACAGAATGCC	
rps17-R	TCCACCGCCACAGTCTTG	
petL-A-F	AGTACTTTAAGAAACATATGTA	
petL-A-R	TCAAATAAGTCGTATTTTGCT	
ndhG-B-F	AAACAGCCTAATACTAATAA	
ndhG-B-R	TATATTATTAAAATAAGAAAAAATCG	
ndhG-C-F	TAATATAATGGATTTGCCTGGACCAATAC	
ndhG-C-R	GGGAAGTAATACCACTCCCAG	
ndhG-D-F	AACCCAATATTTTCAGCCTTTTC	
ndhG-D-R	GAAAAGGCTGAAAATATTGGGTT	
petB-nc-F	TTCATCGATGGTCGGCAAG	
petB-nc-R	TGCGGTCAATACACCCAGAA	

Table S2. **Plastid RNA editing sites affected in the *wtg1* mutant plants.**

All 34 reported editing sites in *Arabidopsis* plastid were detected by bulk sequencing. The affected sites, *petL-50* and *ndhG-5*, are shown in bold.

Editing site	Locus	Editing extent in wild-type (%)	Editing extent in *wtg1* (%)	Related editing factors	References
matK-640	2931	90	90	QED1 (PPR), MORF2, MORF9, ORRM1, OZ1	(Tillich *et al.*, 2005; Takenaka *et al.*, 2012; Sun *et al.*, 2013; Sun *et al.*, 2015; Wagoner *et al.*, 2015)
atpF-92	12707	100	100	AEF1 (PPR), MORF2, MORF9	(Tillich *et al.*, 2005; Chateigner-Boutin & Small, 2007; Takenaka *et al.*, 2012)
rpoC1-488	21806	30	30	DOT4 (PPR), MORF2, MORF8, MORF9, OZ1, PPO1	(Tillich *et al.*, 2005; Bentolila *et al.*, 2012; Takenaka *et al.*, 2012; Hayes *et al.*, 2013; Zhang *et al.*, 2014; Sun *et al.*, 2015)
rpoB-2432	23898	90	90	QED1 (PPR), MORF2, MORF8, MORF9, ORRM1, OZ1	(Tillich *et al.*, 2005; Bentolila *et al.*, 2012; Takenaka *et al.*, 2012; Sun *et al.*, 2013; Sun *et al.*, 2015; Wagoner *et al.*, 2015)
rpoB-551	25779	90	90	CRR22 (PPR), MORF2, MORF9, ORRM1, OZ1	(Tillich *et al.*, 2005; Okuda *et al.*, 2009; Takenaka *et al.*, 2012; Zhang *et al.*, 2014; Sun *et al.*, 2015)
rpoB-338	25992	90	90	YS1 (PPR), MORF2, MORF9, OZ1, PPO1	(Tillich *et al.*, 2005; Zhou *et al.*, 2009; Takenaka *et al.*, 2012; Zhang *et al.*, 2014; Sun *et al.*, 2015)

psbZ-50	35800	100	100	OTP84 (PPR), MORF2, MORF9, OZ1, PPO1	(Chateigner-Boutin & Small, 2007; Hammani *et al.*, 2009; Takenaka *et al.*, 2012; Zhang *et al.*, 2014; Sun *et al.*, 2015)
rps14-80	37161	100	100	MORF2, MORF9	(Tillich *et al.*, 2005; Takenaka *et al.*, 2012)
rps14-149	37092	100	100	OTP86 (PPR), MORF2, MORF9	(Tillich *et al.*, 2005; Hammani *et al.*, 2009; Takenaka *et al.*, 2012)
accD-794	57868	100	100	RARE1/AtECB2 (PPR), MORF2, MORF8, MORF9, OZ1	(Tillich *et al.*, 2005; Robbins *et al.*, 2009; Yu *et al.*, 2009; Tseng *et al.*, 2010; Bentolila *et al.*, 2012; Takenaka *et al.*, 2012; Sun *et al.*, 2015; Wagoner *et al.*, 2015)
accD-3'UTR	58642	80	80	QED1/AtECB2 (PPR), MORF2, MORF9, ORRM1, OZ1	(Chateigner-Boutin & Small, 2007; Robbins *et al.*, 2009; Tseng *et al.*, 2010; Takenaka *et al.*, 2012; Sun *et al.*, 2013; Sun *et al.*, 2015; Wagoner *et al.*, 2015)
psbF-77	63985	100	100	LPA66 (PPR), MORF2, MORF9, OZ1	(Tillich *et al.*, 2005; Cai *et al.*, 2009; Takenaka *et al.*, 2012; Sun *et al.*, 2015)
psbE-214	64109	100	100	CREF3 (PPR), MORF2	(Tillich *et al.*, 2005; Takenaka *et al.*, 2012; Yagi *et al.*, 2013)
***petL*-5**	65716	100	40	MORF2, MORF8, MORF9, OZ1, PPO1	(Tillich *et al.*, 2005; Bentolila *et al.*, 2012; Takenaka *et al.*, 2012; Zhang *et al.*, 2014; Sun *et al.*, 2015)
rps12-i58	69553	30	30	OTP81/QED1 (PPR), MORF2, MORF8, MORF9, ORRM1, OZ1	(Chateigner-Boutin & Small, 2007; Hammani *et al.*, 2009; Bentolila *et al.*, 2012; Takenaka *et al.*, 2012; Sun *et al.*, 2013; Sun *et al.*, 2015; Wagoner *et al.*, 2015)

clpP-559	69942	80	80	CLB19 (PPR), MORF2, MORF9, ORRM1, OZ1	(Tillich *et al.*, 2005; Chateigner-Boutin *et al.*, 2008; Takenaka *et al.*, 2012; Sun *et al.*, 2013; Sun *et al.*, 2015)
rpoA-200	78691	80	80	CLB19 (PPR), MORF2, MORF9, OZ1	(Chateigner-Boutin & Small, 2007; Chateigner-Boutin *et al.*, 2008; Takenaka *et al.*, 2012; Sun *et al.*, 2015)
rpl23-89	86055	80	80	OTP80 (PPR), MORF2, MORF9, OZ1, PPO1	(Chateigner-Boutin & Small, 2007; Hammani *et al.*, 2009; Takenaka *et al.*, 2012; Zhang *et al.*, 2014; Sun *et al.*, 2015)
ndhB-1481	94999	90	90	OTP84 (PPR), MORF2, MORF8, MORF9, OZ1, PPO1	(Tillich *et al.*, 2005; Hammani *et al.*, 2009; Bentolila *et al.*, 2012; Takenaka *et al.*, 2012; Zhang *et al.*, 2014; Sun *et al.*, 2015)
ndhB-1255	95225	100	100	CREF7 (PPR), MORF2, MORF9, ORRM1, OZ1, PPO1	(Tillich *et al.*, 2005; Takenaka *et al.*, 2012; Sun *et al.*, 2013; Yagi *et al.*, 2013; Zhang *et al.*, 2014; Sun *et al.*, 2015)
ndhB-872	95608	90	90	QED1 (PPR), MORF2, MORF9, ORRM1, OZ1, PPO1	(Tillich *et al.*, 2005; Takenaka *et al.*, 2012; Sun *et al.*, 2013; Zhang *et al.*, 2014; Sun *et al.*, 2015; Wagoner *et al.*, 2015)
ndhB-836	95644	100	100	OTP82 (PPR), MORF2, MORF8, MORF9, ORRM1, OZ1, PPO1	(Tillich *et al.*, 2005; Bentolila *et al.*, 2012; Takenaka *et al.*, 2012; Hayes *et al.*, 2013; Sun *et al.*, 2013; Zhang *et al.*, 2014; Sun *et al.*, 2015)
ndhB-830	95650	100	100	ELI1 (PPR), MORF2, MORF8, MORF9, ORRM1, OZ1, PPO1	(Tillich *et al.*, 2005; Okuda *et al.*, 2009; Bentolila *et al.*, 2012; Takenaka *et al.*, 2012; Sun *et al.*, 2013; Zhang *et al.*, 2014; Sun *et al.*, 2015)

ndhB-746	96419	100	100	CRR22 (PPR), MORF2, MORF8, MORF9, OZ1	(Tillich *et al.*, 2005; Bentolila *et al.*, 2012; Takenaka *et al.*, 2012; Zhang *et al.*, 2014; Sun *et al.*, 2015)
ndhB-586	96579	100	100	MORF2, MORF8, MORF9, ORRM1, OZ1, PPO1	(Tillich *et al.*, 2005; Bentolila *et al.*, 2012; Takenaka *et al.*, 2012; Sun *et al.*, 2013; Zhang *et al.*, 2014; Sun *et al.*, 2015)
ndhB-467	96698	90	90	CRR28 (PPR), MORF2, MORF8, MORF9, ORRM1, OZ1, PPO1	(Tillich *et al.*, 2005; Okuda *et al.*, 2009; Bentolila *et al.*, 2012; Takenaka *et al.*, 2012; Sun *et al.*, 2013; Zhang *et al.*, 2014; Sun *et al.*, 2015)
ndhB-149	97016	100	100	MORF2, MORF9, PPO1	(Tillich *et al.*, 2005; Takenaka *et al.*, 2012; Zhang *et al.*, 2014)
ndhF-290	112349	100	100	OTP84/AtECB2 (PPR), MORF2, MORF8, MORF9, OZ1, PPO1	(Tillich *et al.*, 2005; Hammani *et al.*, 2009; Tseng *et al.*, 2010; Bentolila *et al.*, 2012; Takenaka *et al.*, 2012; Zhang *et al.*, 2014; Sun *et al.*, 2015)
ndhD-887	116281	90	90	CRR22 (PPR), MORF2, MORF9, ORRM1, OZ1, PPO1	(Tillich *et al.*, 2005; Okuda *et al.*, 2009; Takenaka *et al.*, 2012; Sun *et al.*, 2013; Zhang *et al.*, 2014; Sun *et al.*, 2015)
ndhD-878	116290	80	80	CRR28 (PPR), MORF2, MORF9, ORRM1, OZ1, PPO1	(Tillich *et al.*, 2005; Okuda *et al.*, 2009; Takenaka *et al.*, 2012; Sun *et al.*, 2013; Zhang *et al.*, 2014; Sun *et al.*, 2015)
ndhD-674	116494	100	100	OTP85 (PPR), MORF2, MORF9, ORRM1, OZ1, PPO1	(Tillich *et al.*, 2005; Hammani *et al.*, 2009; Takenaka *et al.*, 2012; Sun *et al.*, 2013; Zhang *et al.*, 2014; Sun *et al.*, 2015)

ndhD-383	116785	100	100	CRR21 (PPR), MORF2, MORF8, MORF9, PPO1	(Tillich *et al.*, 2005; Okuda *et al.*, 2007; Bentolila *et al.*, 2012; Takenaka *et al.*, 2012; Zhang *et al.*, 2014)
ndhD-2	117166	50	50	CRR4 (PPR), MORF2, MORF8, MORF9, OZ1, PPO1	(Kotera *et al.*, 2005; Tillich *et al.*, 2005; Okuda *et al.*, 2006; Bentolila *et al.*, 2012; Takenaka *et al.*, 2012; Zhang *et al.*, 2014; Sun *et al.*, 2015)
***ndhG*-50**	118858	100	50	OTP82 (PPR), MORF2, MORF9, ORRM1, OZ1, PPO1	(Tillich *et al.*, 2005; Okuda *et al.*, 2010; Takenaka *et al.*, 2012; Sun *et al.*, 2013; Zhang *et al.*, 2014; Sun *et al.*, 2015)

Table S3 *Trans*-factors involved in *petL-5* and/or *ndhG-50* editing

Trans-factor	Gene ID	Gene family	Editing extent in the mutant (%)		Editing level change mutant :: WT		Reference
			petL -5	*ndhG -50*	*petL -5*	*ndhG -50*	
OTP82	AT1G08070	PPR	n/a	1	n/a	-99%	(Hammani *et al.*, 2009; Okuda *et al.*, 2010; Kindgren *et al.*, 2015)
MORF2	AT2G33430	MORF	65	40	-35%	-60%	(Takenaka *et al.*, 2012)
MORF8	AT3G15000	MORF	35	72	-60%	-6%	(Bentolila *et al.*, 2012)
MORF9	AT1G11430	MORF	0	0	-100%	-100%	(Takenaka *et al.*, 2012)
ORRM1	AT3G20930	ORRM	66	8	+1%	-94%	(Sun *et al.*, 2013)
PPO1	AT4G01690	Protoporphyrinogen oxidase	65	85	-19%	-15%	(Zhang *et al.*, 2014)
OZ1	AT5G17790	RanBP2-type zinc finger	45	0	-42%	-100%	(Sun *et al.*, 2015)

+ indicates increased editing extent compared to that of wild-type plants; - indicates decreased editing extent compared to that of wild-type plants. n/a, not assayed.

References

Bentolila S, Heller WP, Sun T, Babina AM, Friso G, van Wijk KJ, Hanson MR. 2012. RIP1, a member of an Arabidopsis protein family, interacts with the protein RARE1 and broadly affects RNA editing. Proceedings of the National Academy of Sciences, USA **109**, E1453-1461.

Cai W, Ji D, Peng L, Guo J, Ma J, Zou M, Lu C, Zhang L. 2009. LPA66 is required for editing psbF chloroplast transcripts in Arabidopsis. Plant Physiology **150**, 1260-1271.

Chateigner-Boutin AL, Ramos-Vega M, Guevara-Garcia A, *et al*. 2008. CLB19, a pentatricopeptide repeat protein required for editing of rpoA and clpP chloroplast transcripts. The Plant Journal **56**, 590-602.

Chateigner-Boutin AL, Small I. 2007. A rapid high-throughput method for the detection and quantification of RNA editing based on high-resolution melting of amplicons. Nucleic Acids Research **35**, e114.

Hammani K, Okuda K, Tanz SK, Chateigner-Boutin AL, Shikanai T, Small I. 2009. A study of new Arabidopsis chloroplast RNA editing mutants reveals general features of editing factors and their target sites. The Plant Cell **21**, 3686-3699.

Hayes ML, Giang K, Berhane B, Mulligan RM. 2013. Identification of two pentatricopeptide repeat genes required for RNA editing and zinc binding by C-terminal cytidine deaminase-like domains. The Journal of Biological Chemistry **288**, 36519-36529.

Kindgren P, Yap A, Bond CS, Small I. 2015. Predictable alteration of sequence recognition by RNA editing factors from Arabidopsis. The Plant Cell **27**, 403-416.

Kotera E, Tasaka M, Shikanai T. 2005. A pentatricopeptide repeat protein is essential for RNA editing in chloroplasts. Nature **433**, 326-330.

Okuda K, Chateigner-Boutin AL, Nakamura T, Delannoy E, Sugita M, Myouga F, Motohashi R, Shinozaki K, Small I, Shikanai T. 2009. Pentatricopeptide repeat proteins with the DYW motif have distinct molecular functions in RNA editing and RNA cleavage in Arabidopsis chloroplasts. The Plant Cell **21**, 146-156.

Okuda K, Hammani K, Tanz SK, Peng L, Fukao Y, Myouga F, Motohashi R, Shinozaki K, Small I, Shikanai T. 2010. The pentatricopeptide repeat protein OTP82 is required for RNA editing of plastid ndhB and ndhG transcripts. The Plant Journal **61**, 339-349.

Okuda K, Myouga F, Motohashi R, Shinozaki K, Shikanai T. 2007. Conserved domain structure of pentatricopeptide repeat proteins involved in chloroplast RNA editing. Proceedings

of the National Academy of Sciences, USA **104**, 8178-8183.

Okuda K, Nakamura T, Sugita M, Shimizu T, Shikanai T. 2006. A pentatricopeptide repeat protein is a site recognition factor in chloroplast RNA editing. The Journal of Biological Chemistry **281**, 37661-37667.

Robbins JC, Heller WP, Hanson MR. 2009. A comparative genomics approach identifies a PPR-DYW protein that is essential for C-to-U editing of the Arabidopsis chloroplast accD transcript. RNA **15**(6): 1142-1153.

Sun T, Germain A, Giloteaux L, Hammani K, Barkan A, Hanson MR, Bentolila S. 2013. An RNA recognition motif-containing protein is required for plastid RNA editing in Arabidopsis and maize. Proceedings of the National Academy of Sciences, USA **110**, E1169-1178.

Sun T, Shi X, Friso G, Van Wijk K, Bentolila S, Hanson MR. 2015. A zinc finger motif-containing protein is essential for chloroplast RNA editing. PLoS Genetics **11**, e1005028.

Takenaka M, Zehrmann A, Verbitskiy D, Kugelmann M, Hartel B, Brennicke A. 2012. Multiple organellar RNA editing factor (MORF) family proteins are required for RNA editing in mitochondria and plastids of plants. Proceedings of the National Academy of Sciences, USA **109**, 5104-5109.

Tillich M, Funk HT, Schmitz-Linneweber C, Poltnigg P, Sabater B, Martin M, Maier RM. 2005. Editing of plastid RNA in Arabidopsis thaliana ecotypes. The Plant Journal **43**, 708-715.

Tseng CC, Sung TY, Li YC, Hsu SJ, Lin CL, Hsieh MH. 2010. Editing of accD and ndhF chloroplast transcripts is partially affected in the Arabidopsis vanilla cream1 mutant. Plant Molecular Biology **73**(3): 309-323.

Wagoner JA, Sun T, Lin L, Hanson MR. 2015. Cytidine deaminase motifs within the DYW domain of two pentatricopeptide repeat-containing proteins are required for site-specific chloroplast RNA editing. The Journal of Biological Chemistry **290**, 2957-2968.

Yagi Y, Tachikawa M, Noguchi H, Satoh S, Obokata J, Nakamura T. 2013. Pentatricopeptide repeat proteins involved in plant organellar RNA editing. RNA Biology **10**, 1419-1425.

Yu QB, Jiang Y, Chong K, Yang ZN. 2009. AtECB2, a pentatricopeptide repeat protein, is required for chloroplast transcript accD RNA editing and early chloroplast biogenesis in Arabidopsis thaliana. The Plant Journal **59**, 1011-1023.

Zhang F, Tang W, Hedtke B, Zhong L, Liu L, Peng L, Lu C, Grimm B, Lin R. 2014. Tetrapyrrole biosynthetic enzyme protoporphyrinogen IX oxidase 1 is required for plastid RNA

editing. Proceedings of the National Academy of Sciences, USA **111**, 2023-2028.

Zhou W, Cheng Y, Yap A, Chateigner-Boutin AL, Delannoy E, Hammani K, Small I, Huang J. 2009. The Arabidopsis gene YS1 encoding a DYW protein is required for editing of rpoB transcripts and the rapid development of chloroplasts during early growth. The Plant Journal **58**, 82-96.
